# Pharmacological evaluation of newly synthesized organotin IV complex for antiulcer potential

**DOI:** 10.1186/s40360-022-00596-0

**Published:** 2022-07-29

**Authors:** Syed Azmatullah, Arif-ullah Khan, Neelam Gul Qazi, Humaira Nadeem, Nadeem Irshad

**Affiliations:** 1 Department of Pharmacology, Riphah Institute of Pharmaceutical Sciences, Riphah International University, Islamabad, Pakistan; 2 Department of Pharmaceutical Chemistry, Riphah Institute of Pharmaceutical Sciences, Riphah International University, Islamabad, Pakistan; 3 Department of Pharmacy, Quaid i Azam University, Islamabad, Pakistan

**Keywords:** 2E, 2′E) dibutylstannanediyl bis(4-((4-nitrophenyl) amino)-4-oxobut-2-enoate, Anti-gastric ulcer, Anti-*H. pylori*, H^+^/K^+^-ATPase inhibition, Anti-oxidant, Anti-inflammatory

## Abstract

The present study aims to investigate the newly synthesized organotin (IV) complex (2E, 2′E) dibutylstannanediyl bis (4-(4-nitrophenyl) amino)-4-oxobut-2-enoate (DTN) for its anti-ulcer potential. Characterization performed by carbon nuclear magnetic resonance spectroscopy proved that all values are in the expected ranges of the new compound. Gastroprotective activity of DTN was evaluated through *in-silico,* anti-*H. pylori,* in-vitro*,* in-vivo, and ex-vivo proteomic analysis. *In-silico* analysis shows that DTN possess stable binding with protein targets involved in gastric ulcer pathophysiology. DTN exhibited an inhibitory effect against 2,2-diphenyl-1-picrylhydrazyl, *H. pylori* and hydrogen potassium ATPase (H^+^/K^+^-ATPase). The antiulcer activity was performed using an ethanol-induced gastric ulcer model in rats. Anti-oxidant profile of DTN showed a significant increase in glutathione-S-transferase, glutathione and catalase levels whereas lipid peroxidation levels were reduced. Histopathological findings confirmed that DTN protected the gastric mucosa of rats. Inflammatory markers tumor necrosis factor-alpha, nuclear factor kappa B, cyclooxygenase-2, interleukin 6 and interleukin-1β were reduced and prostaglandin-E_2_ restored expression of these cytokines in DTN pretreated animals when analyzed by using immunohistochemistry, enzyme-linked immunosorbent assay and western blot techniques. In real-time polymerase chain reaction technique, the expression of H^+^/K^+^-ATPase was downregulated in DTN pretreated group. DTN did not cause any mortality up to 400 mg/Kg. This study indicates that the newly synthesized compound DTN, possess stable binding against selected targets. DTN exhibits a gastro-protective effect, mediated via anti-*H. pylori,* H^+^/K^+^-ATPase inhibition, anti-oxidant and anti-inflammatory pathways, exploring its therapeutic potential in gastric ulcer management.

**Supplementary Information:**

The online version contains supplementary material available at 10.1186/s40360-022-00596-0.

## Introduction

Gastric ulcer is one of the most common chronic gastrointestinal diseases characterized by a significant defect in the mucosal barrier. About 5 to 10% of people were infected and a major public health burden in last two decades [1]. There is an imbalance between offensive mucosal factors such as long-term periodic consumption of non-steroidal anti-inflammatory drugs, smoking, alcohol, infectious agents and stress and defensive mucosal factors specifically prostaglandin levels and antioxidant enzyme activity, which leads to disruption of gastric mucosa thus causing stomach ulcers [2]. *H. pylori* bacteria is considered one of the major causes of gastric ulcers in humans [3]. Hypersecretion of acids and pepsins in the stomach due to overactivation of H^+^/K^+^-ATPase pump where K^+^ pumps in and Na^+^ out and suppression of blood flow in gastric mucosa leads to gastric ulceration [4]. Oxidative stress plays an important role in gastric ulcer formation due to the generation of highly cytotoxic free radicals [5, 6]. On the other hand, ethanol upsets the stomach secretory activity changing the permeability of the cells and disrupting the protective mucus layer which blocks gastric mucosal defense [7].

The ethanol-induced gastric ulcer model resembles gastric ulcer disease in humans [5, 8]. Gastric ulcer leads to gastric pain, blood in stools, nausea, vomiting, heartburn, weight loss and loss of appetite [9]. So the goal is to relieve the pain and prevent ulceration. Multi drugs such as antibiotics, antacids, proton pump inhibitors omeprazole and antihistamine are readily available to treat ulcers. However, major problems are encountered due to the limited efficacy against the gastrointestinal tract and their severe side effects. For example, gynecomastia, hypoacidity, impotence, osteoporotic bone fracture, hypergastrinemia and the risk of heart diseases [10]. Molecular docking is the key method of structured virtual screening and it is still a very active area in research [11]. Thus new candidates who can provide high efficacy and low toxicity are valuable for the prevention and treatment of gastric ulcer.

Organometallic compounds containing Sn-C atoms are linked to the organic moiety direct Sn-C covalent bonds are known to explode with organotin (IV) compounds. The general formula of (RnSnX4-n) where R contains organic matter, X, any anionic group such as like Cl−, OH– etc. and *n* = 1–4. Depending on the number of organic moieties (n) they are connected to the Sn atom the organotin (IV) compounds are classified as RSnX3, R2SnX2, R3SnX and R4Sn: and are called mono, di, tri and tetra organotin (IV) compounds [12]. Tin metallic element strongly affects biochemical orientation of organotin (IV) compounds.

**Fig. 1:**
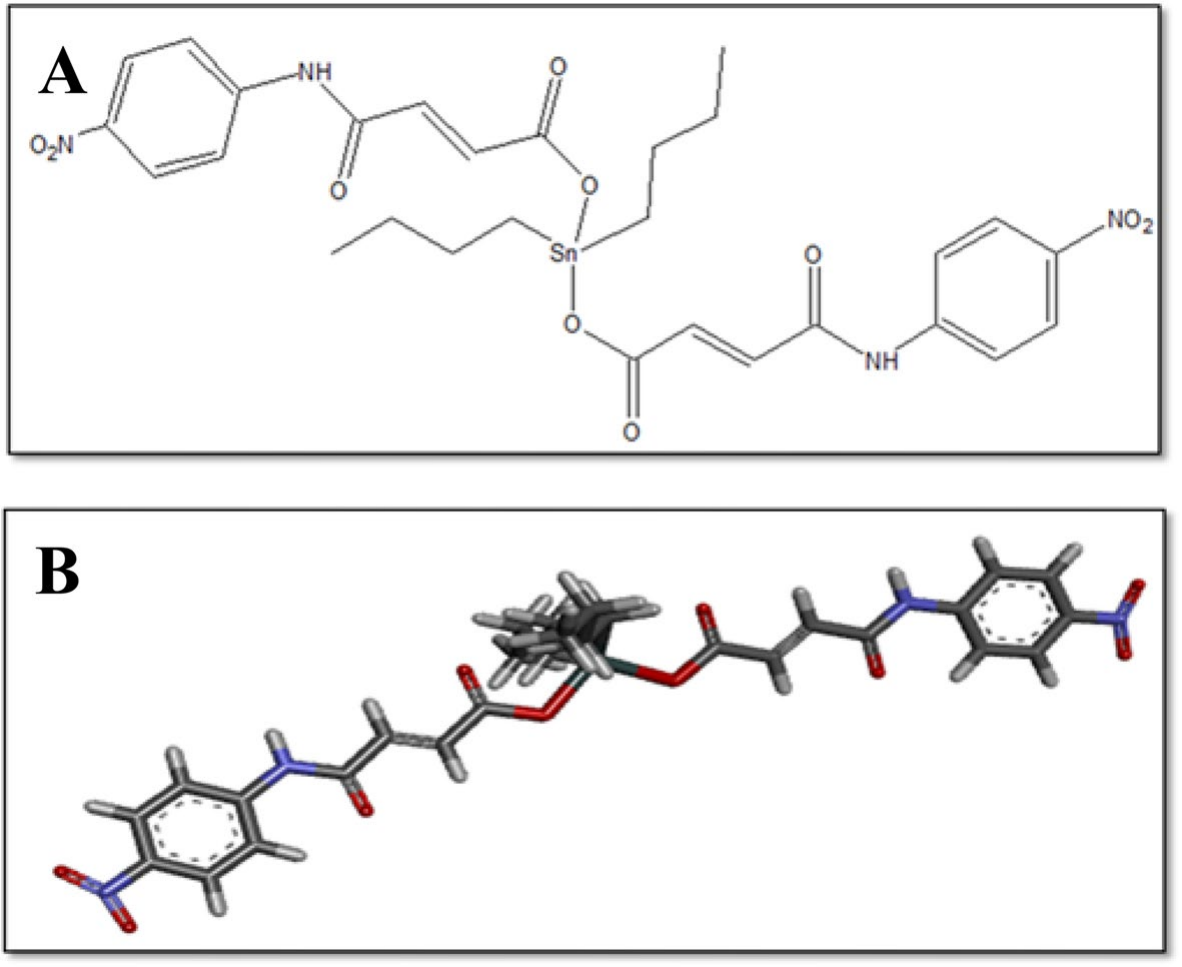
**A** and **B** represents 2D and 3D-structures of 2E,2′E) dibutylstannanediyl bis (4-((4-nitrophenyl) amino)-4-oxobut-2-enoate (DTN)

Organotin (IV) compounds have many applications in non-biological and biological aspects [13]. Organotin (IV) covers the biological aspect of a wide field of medical chemical science due to its structural diversity and extensive treatment applications [14]. Organotin (IV) compounds contains potent anti-tumor, anti-bacterial and anti-fungal activities [15]. Anti-cancer and anti-leishmanial potential of organotin (IV) complexes activity was reported [16]. In particular organotin (IV) has shown significant biological activities such as biocides, cytotoxicity, anti-proliferation, anti-tuberculosis and anti-inflammatory [17, 18]. The aim of this study is evaluation of newly synthesized compound of organotin (IV) complex (2E,2′E) dibutylstannanediyl bis(4-((4-nitrophenyl) amino)-4-oxobut-2-enoate) (DTN) (Fig. 1), for its effectiveness against ethanol-induced gastric ulcer model in rats, using *in*-*silico,* in-vitro*,* in-vivo and ex-vivo proteomic analysis techniques.

## Material and methods

### Chemicals

Dibutyltindichloride, maellicanhydride, 4-Nitro aniline, ethanol, dimethyl sulfoxide (DMSO), chloroform and normal saline acquired from Sigma-Aldrich, Germany. DTN compound was prepared in postgraduate chemistry research laboratory of Riphah Institute of Pharmaceutical Sciences (RIPS) Islamabad, Pakistan. Omeprazole, metronidazole purchased from Barrett Hodgson and Sanofi Aventis. Secondary antibodies procured from Abcam UK. Rat NF-ĸB ELISA kit (Catalog No. E-El-R0676), rat TNF-α ELISA kit (Catalog No. E-El-R0019), rat IL-6 (Catalog No. DY406) and rat IL-1β (Catalog No. ab100704) and rat PGE_2_ ELISA kit (Catalog No. E-El-R0034) were purchased from Elabscience, China. All chemicals were used in experiments analytically approved (99% HPLC grade).

### Animals

Sprague-Dawley rats (180-240 g) of both sex have been used in the experimental process kept in animal house of the Riphah Institute of Pharmaceutical Sciences (RIPS) Islamabad, Pakistan, with organized environment provided (20-25 °C). Experiment trials were performed according to the rules and regulations of Research and Ethics Committee RIPS (Ref. No. REC/RIPS/2021/018) along with the guidelines of “Principles of Laboratory Animal care “.

### Synthesis and characterization

#### 2E, 2′E dibutylstannanediyl bis (4-(4-nitrophenyl) amino)-4-oxobut-2-enoate (DTN)

**Fig. 2:**
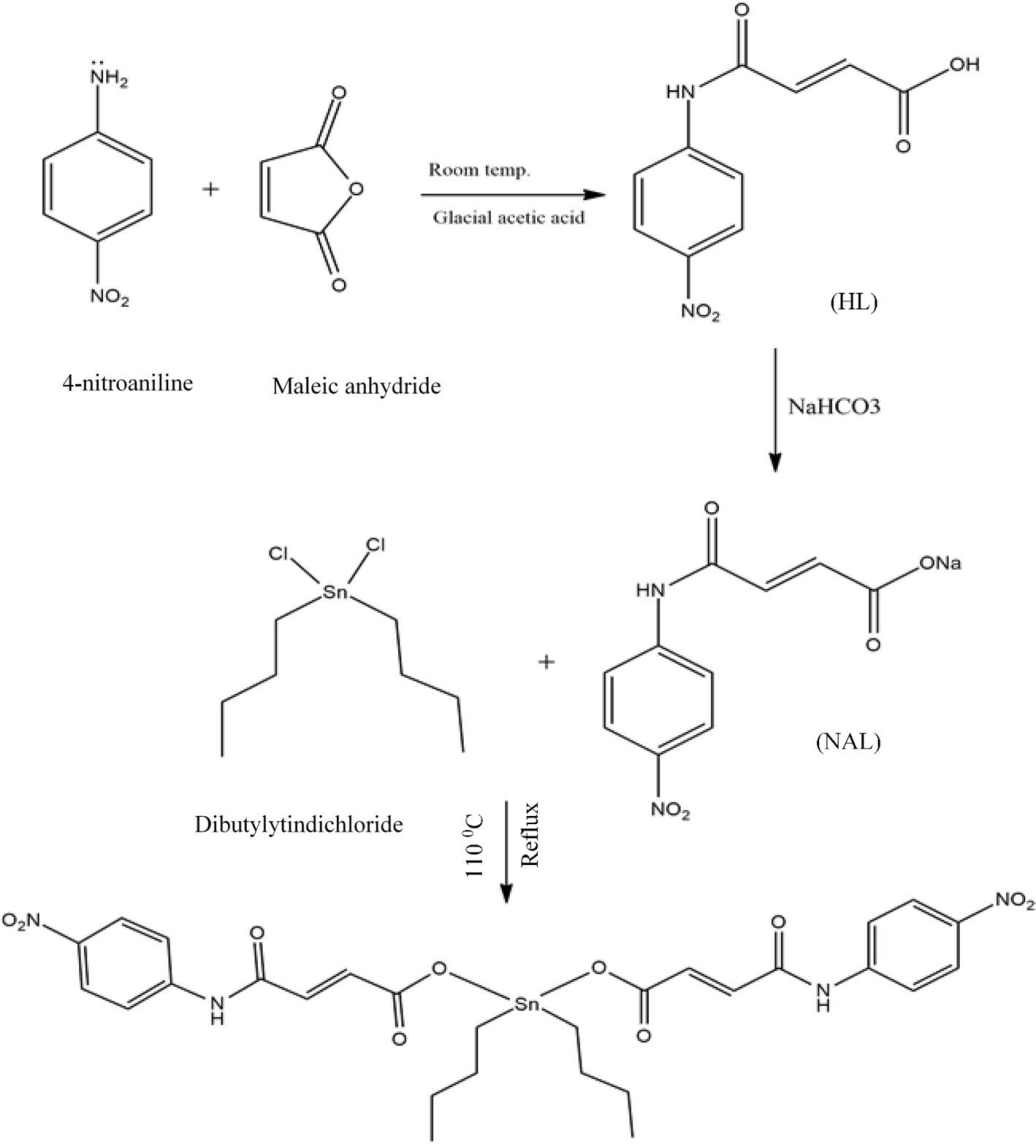
Chemical synthesis of 2E,2′E) dibutylstannanediyl bis(4-(4-nitrophenyl)amino)-4-oxobut-2-enoate (DTN)

Sodium salt of ligand was (NaL) product was prepared by reacting the synthesized carboxylic acid with aqueous solution of (NaHCO_3_) and stirred continuously for 15-20 minutes. In the next step, NaL was reacted with equimolar quantities of dibutyltindichloride in dried toluene solvent and refluxing for 8–12 hrs at temperature 110 °C. TLC was employed to check the reaction progress, after completion reaction mixture was filtered, evaporated in vacuo to get final desired product DTN (Fig. 2) as reported earlier [16]. Carbon-13 nuclear magnetic resonance spectroscopy (^13^CNMR) (Bruker-AM 300spectrophotometer) was employed to determine the composition analysis of new compound [16].

### Computational study

Docking studies were performed in Autodock 4.2 and CDOCKER. The parameter file of Autodock was modified due to presence of metal in ligand structure thus needed parameters which were obtained from the Autodock website (http://autodock.scripps.edu/resources/parameters). The three-dimensional structure (3D) of the newly synthesized test compound DTN was drawn in chem sketch. The 3D structure of standard drugs i.e. omeprazole, phenoxybenzamine, ranitidine, aspirin, meclofenamate, dinoprostone and curcumin was obtained from PubChem. Human protein targets involved in the pathogenesis of gastric ulcer were selected and 3D structures were acquired from the online protein database, Research collaboratory for structural bioinformatics (RCSB) PDB. The selected target proteins were histamine receptor H_2_ (PDB ID: P25021), hydrogen potassium ATPase H^+^/K^+^-ATPase (PDB ID: 5ylu), muscarinic receptor M_1_ (PDB ID: 5CXV), prostaglandin-E2 PGE_2_ (PDB ID: 6AK3), Tumor necrosis factor-alpha TNF-α (PDB ID: 1BKC), nuclear factor kappa B NFĸB (PDB ID: 4Q3J), cycloxygenase-1 COX_1_ (PDB ID: 6Y3C) and cycloxygenase-2 COX_2_ (PDB ID: 5IKQ). Ligand water molecules were removed in discovery studio visualizer (DSV) and H (polar) atoms were added and saved in PDB format. Auto dock tools 1.5.6 and Auto Dock 4.2 docking software were used for molecular docking. The results were analyzed as the atomic contact energy (ACE-value) (Kcal/mol). One best pose with lowest ACE value (kcal/mol) was selected for post-dock analysis via Biovia DSV. By determination of the interactions between ligand and amino acid residues were evaluated through 2D images [19].

C-DOCKER module of DSV was used to crosscheck the docking results of AutoDock. C-DOCKER is an implementation of CHARMm-based docking tool. The receptor is held rigid while the ligands are allowed to flex during the docking process. For each complex pose, the CHARMm energy (interaction energy plus ligand strain) and the interaction energy, which indicate ligand binding affinity are calculated. The crystal water molecules are generally removed in rigid and semi-flexible docking process [20], since the fixed water molecules might affect the formation of receptor-ligand complex. The water molecules were removed and hydrogen atoms were added to the protein. One best pose with lowest CDOCKER interaction energy was selected for post docking analysis.

### DPPH free radical scavenging assay

2,2-diphenyl-1-picrylhydrazyl (DPPH) stock solution was prepared in 100 mL methanol by dissolving 9.2 mg DPPH. The ascorbic acid stock solution was prepared by dissolving (1 mg/mL) DMSO. Four milligrams compound was dissolved in DMSO for the preparation of the stock solution. These samples were tested for their potential of scavenging free radicals. In each well of 96 well microplates, test samples were transferred to the relevant well and then (190 μL) DPPH reagents were added to each well. 200 μg/mL was the final sample concentration. The reaction mixture was incubated in the dark at 37 °C; the absorbance was measured at 517 nm [15]. Free radical scavenging activity was calculated using the formula.

$$\%\,\mathrm{scavenging}\ \mathrm{activity}=\left(1-\mathrm{Abs}/\mathrm{Abc}\ \right)\ \mathrm{x}\ 100$$

Where, Abs (sample absorbance), Abc (Absorbance of negative control). Triple serial dilution were (200, 66.6, 22.2, 7.4 and 2.46 μg/mL) concentrations.

### Anti-*H. pylori* activity

The antibacterial activity of DTN compound against *Helicobacter pylori* (*H. pylori*) was evaluated through disc diffusion method [21]. Three different strains of *H. pylori* j63 (cagA^−^), j196 (cagA^−^) and j107 (cagA^+^) were obtained from the biopsy of the gastric ulcer patient, voluntarily at care endoscopy clinics and labs (Rawalpindi, Pakistan). Biopsies were placed in modified thio campy medium. The plates were incubated at 37 °C under microaerophilic environment. The isolates were identified by morphology and urease test kit. The isolates were kept in sterile McCartney bottles containing 0.2 g / L of cysteine and 20% of glycerol in brain heart infusion BHI broth 80 °C. Frozen clinical isolates were inoculated on Muller-Hinton Agar (MHA) plates. DTN test compound with different concentrations were added onto standard discs and was placed on the MHA plate. After incubation at 37 °C for 3 to 5 days, the zone inhibition was measured for each disk. All tests were performed in triplicate and antibacterial activity was evaluated as the mean of the inhibition diameter (mm). Metronidazole was used as a positive control in the experiment [22].

### Ethanol-induced gastric ulcer

For induction of gastric lesions, after 24 hours of fasting rats were randomly assigned to six different groups (*n* = 5). Group (I) served as a saline control received saline solution of (10 mL/Kg) body weight. Group (II), (III) and (IV) pretreated with DTN at doses of 5, 10 and 30 mg/Kg (p.o) respectively and group (V) received omeprazole (20 mg/Kg) served as standard drug and group (VI) was negative control received absolute ethanol. One hour after all treatments, absolute ethanol (1 mL/100 g) was administered orally to each rat. After 1 hour of ethanol treatment all rats were euthanized by cervical displacement and the stomachs were removed and saline normal solution was used to washed them and the lesion index was estimated by measuring each lesion in mm along its largest curvature scored area of each lesion as measured and marked according to the method previously described by [23]. The percentage of inhibition (% I) had been calculated using the following formula:

$$\%\,\mathrm{I}=\left(\mathrm{USc}-\mathrm{USt}\right)\ \mathrm{x}\ 100/\mathrm{USc}\Big)$$

Where USc = surface area ulcer of control and USt = surface area ulcer of treatment drug group.

### H^+^/K^+^-ATPase inhibitory assay

DTN inhibitory effect on rat gastric H^+^/K^+^-ATPase was analyzed using commercially available calorimetric H^+^/K^+^-ATPase activity test screening kit (Catalog No E-BC-K122-S ElabScience USA). Gastric-tissues kept in biofreezer (− 80 °C) were homogenized using Silent Crusher M (heidolp). The homogenate was then centrifuged for 10 minutes at 3500 rpm and the supernatant was separated. Supernatant was analyzed for release of inorganic phosphate after ATP hydrolysis spectrophotometrically at 660 nm. One ATPase activity unit has been described as one micro mole of inorganic phosphorus released by ATP hydrolysis through ATPase 1 mg per hour tissue protein. It was then expressed as μmol pi/mg prot/hour [24].

### Anti-oxidant profile

Gastric tissue isolated from the rat was homogenized and then centrifuged at 1500 rpm for 30 min. and the supernatant was collected. Supernatant was then estimated for glutathione (GSH), catalase, glutathione-S-transferase (GST) and lipid peroxidation (LPO) levels. GSH levels were determined by oxidation of GSH and DTNP which gave a yellow end product. The absorbance of 2-nitro-5-thiobenzoic acid was calculated at 412 nm with the help of a GSH microplate reader. Values expressed in μmoles/mg of proteins GST activity calculated by extinction coefficient of the product formed and expressed in μmole/mg of CDNB conjugate/min/mg of protein. The GST level was determined by the formation of CDNB conjugate and measured its absorbance at 340 nm. Degradation of H2O2 measured in the presence of catalase at 240 nm absorbance was measured using a catalase microplate reader. The LPO level was analyzed by its resultant end product called malondialdehyde (MDA). Microplate reader was used to measure the absorbance at wavelength of 532 nm where quantitative measurement of LPO was expressed in TBARS nmoles/min/mg of protein [25].

### Hematoxylin and Eosin (H&E) staining

Tissue sections were deparaffinized with absolute xylene (100%) on coated slides and then rehydrated with ethyl alcohol (from 100% (absolute) to 70%). The slides were cleaned with distilled water and immersed for 10 min in hematoxylin. The slides were then placed under running water for 10 minutes in glass jar and treated with 1% HCl and 1% ammonia water. The slides were added for 5–10 min to eosin solution. After due time, the slides were washed in water and they are air dried for some time. The dried slides were dehydrated in graded ethyl alcohol (70, 95, and 100%). The slides were cleaned with xylene and were mounted with a glass cover slip. Images of slides were taken using a light microscope (Olympus, Japan) and analyzed through Image J, a computer-based program, while focusing thoroughly on gastric cell size and shape, inflamed infiltrated cells, and vacuolation. The TIF images were arranged to the same threshold intensity for all groups and examined in the GraphPad Prism.8 [25].

### Immunohistochemistry (IHC)

Immunohistochemical analysis was performed as described previously by [22]. After de-paraffinization, slides were handled for antigen retrieval step (enzymatic method) and then wash out with PBS. The endogenous peroxidase was quenched in methanol for 10 min by applying 3% hydrogen peroxide (H2O2). The slides were incubated with 5% normal goat serum containing 0.1% Triton X-100. After blocking, the slides were incubated overnight with mouse anti-TNF-α, p-NFκB and mouse anti-COX-2 antibodies (dilution 1:100, Santa Cruz Biotechnology). The following morning, after cleaning with 0.1 M PBS, slides were handled for incubation in biotinylated secondary antibody (dilution 1:50) according to the primary antibody origin and serum used. After treatment with secondary antibody, slides were incubated with ABC Elite kit (Santa Cruz Biotechnology) in a humidified chamber for 1 hour. Slides were cleaned with 0.1 M PBS, stained in DAB solution, rinsed with distilled water, dehydrated in a graded ethanol series, settled in xylene and cover-slipped in a mounting medium. TIF images were taken by using a light microscope. Image J software was used for the quantitative determination of hyperactivated p-NFκB, COX-2 and TNF-α by optimizing the background of images according to the threshold intensity and analyzing p-NFκB, COX-2 and TNF-α positive cells at the same threshold intensity for all groups. The intensity is expressed as the relative integrated density of the samples relative to the saline [25].

### Enzyme-linked immunosorbent assay (ELISA)

ELISA of p-NFĸB, prostaglandins E2 (PGE_2_), TNF-α, interleukin 6 (IL-6) and interleukin 1 beta (IL-1β) was performed following the manufacturer’s (Elabscience) instructions. Stomach tissues were homogenized using Silent crusher-M (Heidolph) at 15 rpm × 1000. The supernatant was collected after centrifugation at (1350 X rpm for 15 minutes). With the BCA method, the total protein concentration in each group was determined. In brief, the protein samples were treated with the corresponding antibodies provided by kit. By using an ELISA microplate reader, the concentration of p-NFĸB (Catalog No. E-El-R0676) prostaglandins E_2_ (PGE_2_) (Catalog No. E-El-R0034), and TNF-α (Catalog No. E-El-R0019), IL-6 (Catalog No. DY406) and IL-1 (Catalog No. ab100704) determined. All measures were taken in triplicates [25].

### Western blot

Gastric tissues were lysed in a buffer and homogenized for western blot analysis. Using a bicinchoninic acid (BCA) protein assay kit protein concentration was measured. The 30 μg protein homogenate was fixed onto 12% sodium dodecyl sulfate-polyacrylamide gel electrophoresis and shifted to a polyvinylidene fluoride membrane. Membranes were incubated with primary antibodies, such as p-NFκB and TNF-α, overnight at 4 °C then blocked with 5% bovine serum albumin for 1 h at room temperature. After cleaning three times using tris-buffered saline with 0.1% Tween 20, the membranes proceeded with a 1:1000 dilution of secondary antibodies, such as goat, anti-rabbit for 90 min at room temperature. To visualize the immuno-reactive bands, an enhanced western blotting substrate kit was used. Using Image J software, densitometry evaluated the quantification of protein expression [25].

### Real time polymerase chain reaction (RT-PCR)

According to the manufacturer’s instructions, total ribonucleic acid (RNA) was extracted from the gastric tissues, by using the Trizol method. The first-strand of cDNA was generated from 1 to 2 μg of total RNA by a reverse transcriptase enzyme mix on a PCR thermocycler. The mRNA expression normalized to the expression of β-Actin as a house-keeping gene by 2^-∆∆^CT method with slight modification [25]. Primer sequences for β-Actin and H^+^/K^+^-ATPase were CCCGCGAGTACAACCTTCT (forward) and CGTCATCCATGGCGAACT (reverse) and TATGAATTGTACTCAGTGGA (forward) and TGGTCTGGTACTTCTGCT (reverse), respectively.

### Acute toxicity

Toxicity studies of DTN was performed, which defines the lethal dose versus the non-lethal dose of the test compound in the animal model. Using the acute toxicity model rats were divided into two groups of five rats each. Control group treated with normal saline (10 mL/Kg). By using increasing doses of test compound, the test was performed DTN (50–400 mg/Kg) administered orally in normal saline (10 mL/Kg). Forty-eight hours after treatment the rats were observed for mortality [25].

### Statistical analysis

Results data were calculated with mean ± standard error mean of sample (mean ± SEM)). Image J software of (NIH) was used for morphological data analysis. Results were computed by using of one-way analysis of variance (ANOVA) with applied post hoc turkey’s test by using software (Graph pad prism version 8). Level of significance was considered *P* < 0.05.

## Results

### Chemical characterization

A newly synthesized compound of organotin (IV), chemical formula is C_28_H_32_N_4_O_10_Sn and ^13^C NMR confirmed the structure of synthesized compound as 14.0 (CH_3_), 26.1(CH_2_ butyl), 125.4(C=C), 142.6(C=C), 112.8, 119.3, 126.8, 131.4, 136.0, 145.8(Aryl C), 156.1(C=O), 164.8(C=O). Butyl carbons were observed upfield at 14.0-26.1 ppm. Alkenyl carbon atoms were observed downfield at 125.4 and 142.6 ppm respectively, the most downfield signals were attributed to carbonyl groups of ester and amide moieties. All the aryl carbons resonated in the expected ranges.

### In-silico analysis

DTN compound showed significant binding to various protein receptors when evaluated using AD and CD. Table 1 summarizes atomic contact energy values (ACE-values) (Kcal/mol), residues include H-bonding, π-π bonding and others hydrophobic interaction of the best dock poses of DTN and standard drugs against targets H^+^/K^+^-ATPase pump, muscarinic receptor M_1_, histamine receptor H_2_, COX_1_, COX_2_, PGE_2_, NFĸB and TNF-α using AD and CD (ACE and H-bonding only). Figures (S1 to S8 and S1A to S8A) represents the 2D-view interactions of DTN and standard drugs with their targets through AD and CD respectively. Against the H^+^/K^+^-ATPase pump, through AD and CD, DTN showed an (ACE-value) of − 9.12 and − 9.88 (Kcal/mol) and formed 4 and 1 H-bonds and 5 hydrophobic interactions and standard drug omeprazole showed (ACE-value) -8.2 and − 11.63 (Kcal/mol) formed 2 and zero H-bonds and 6 hydrophobic interactions respectively. Using AD and CD, DTN showed an (ACE-value) -9.44 and − 16.39 (Kcal/mol) against M_1_ receptor and formed 5 and zero H-bonds and 6 hydrophobic interaction and standard drug phenoxybenzamine showed (ACE-value) -8.5 and − 13.38 (Kcal/mol) formed 1 π-π bond and 5 hydrophobic interactions. By employing AD and CD, DTN showed (ACE-value) against the H_2_ receptor formed − 7.10 and − 10.37 (Kcal/mol) and formed 2 and 1 H-bonds and 5 hydrophobic interactions and standard drug ranitidine showed (ACE-value) -6.9 and − 15 (Kcal/mol) formed 1 and zero H-bond and 5 hydrophobic interactions respectively. Using AD and CD against COX_1_, DTN showed (ACE-value) -6.46 and − 6.95 (Kcal/mol) and formed 2 and 2 H-bonds and 5 hydrophobic interaction and standard drug aspirin showed (ACE-value) -6.2 and − 6.61 (Kcal/mol) formed 3 and zero H-bonds and 2 hydrophobic interactions. Against COX_2_ target by using AD and CD, DTN showed an (ACE-value) of − 10.64 and − 13.89 (Kcal/mol) and formed 3 and 2 H-bonds and 1 π-π bond and 3 hydrophobic interactions and standard drug meclofinamate showed (ACE-value) -8.42 and − 8.94 (Kcal/mol) formed 4 and 2 H-bonds and 5 hydrophobic interactions. Against PGE_2_ target by employing AD and CD, the DTN represents (ACE-value) -8.98 and − 18.63 (Kcal/mol) and formed the 2 and 2 H-bonds, 1 π-π bond and 5 hydrophobic interactions and standard drug dinopristone showed (ACE-value) -8.24 and − 14.82 (Kcal/mol) formed 5 and 1 H-bonds and 1 hydrophobic interaction. Against NFĸB target through AD and CD, DTN showed an (ACE-value) -4.03 and − 5.64 (Kcal/mol) and formed 3 and 2 H-bonds 1 π-π bond and 3 hydrophobic interactions and standard drug curcumin showed (ACE-value) -7.13 and − 8.54 (Kcal/mol) formed 4 and 1 H-bonds and 3 hydrophobic interactions. Using AD and CD against TNF-α receptor, DTN showed (ACE-value) -8.06 and − 15.6 (Kcal/mol) and formed 5 and 1 H-bonds and 5 hydrophobic interactions and standard drug aspirin showed (ACE-value) -5.20 and − 6.13 (Kcal/mol) formed 4 and zero H-bonds and 1 hydrophobic interaction respectively.

**Table 1:** Best pose dock analysis showing atomic contact energy (ACE) value (Kcal/mol), hydrogen bonds, π-π bonds and hydrophobic interactions formed by 2E,2′E) dibutylstannanediyl bis(4-((4-nitrophenyl)amino)-4-oxobut-2-enoate (DTN) and standard drugs with targets: hydrogen potassium ATPase (H^+^/K^+^-ATPase), muscarinic receptor (M_1_) histaminergic receptor (H_2_), cyclooxygenase-1 (COX_1_), cyclooxygenase-2 (COX_2_), prostaglandin-E2 (PGE_2_), nuclear factor kappa-B (NFκB), and tumor necrosis factor (TNF-α)

DTN								Standard drugs						
Target Protein	ID Code	ACE-value AD	H-bonds AD	Residues forming H-bonds AD	π-π bonds AD	π-π bonds residue AD	Hydrophobic interaction Residues AD	Drug name	ACE-value AD	H-bonds AD	Residues forming H-bonds AD	π-π bonds AD	Residues forming π-π bonds AD	Hydrophobic interactions residues AD
		CD	CD	CD	CD	CD	CD		CD	CD	CD	CD	CD	CD
H^+^/K^+^ ATPase	5YLU	−9.12	4	ASP-137 ARG-328 TYR-799 SYS-813	–	–	LEU-141 ALA-335 VAL-338 LEU-809 GLY-812	Omeprazole	−8.2	2	CYS − 813 ASP − 137	–	–	ILE-816 LEU-141 LEU-796 TYR-799 ALA-335 ALA-339
		−9.88	1	TYR-802	–	–	–		− 11.63	–	–	–	–	–
M_1_	5CXV	−9.44	5	TYR-106 ILE-180 THR-189 THR-192 TYR-381	–	–	TYR-82 LEU-86 ALA-193 GLU-401 TYR-404 GLY-89	Phenoxy- benzamine	−8.5	–	–	1	TYR-404	VAL-113 TRP-387 TYR-381 CYS-407 ALA-196
		−16.39	–	–	–	–	–		−13.38	–	–	–	–	–
H_2_	H2P25021	−7.10	2	ARG-116 ARG-293	–	–	LYS-231 ILE-205 LEU-236 ALA-232 ARG-228	Ranitidine	−6.9	1	LYS-231	–	–	ARG-116 ASN-54 ASN-292 TYR-288 TYR-202
		−10.37	1	LYS-231	–	–	–		−15	–	–	–	–	–
Cox_1_	6Y3C	−6.46	2	ARG-581 VAL-582	–	–	SER-87 HIS-90 HIS-95 GLY-193 PRO-514	Aspirin	−6.2	3	SER-121 GLN-372 LYS-532	–	–	THR-118 ASN-122
		−6.95	2	ARG-581 PRO-191	–	–	–	–	−6.61	–	–	–	–	–
Cox_2_	5IKQ	−10.64	3	GLN-370 GLN-372 LYS-532	1	PRO-543	SER-126 PHE-368 ALA-544	Meclofinamate	−8.42	4	GLN-372 LYS-532 SER-121 ILE-124	–	–	TYR-374 PRO-543 ALA-544 SER-126 HIS-122
		−13.89	2	GLN-372 GLN-372	–	–	–		−8.94	2	GLN-372 PHE-372	–	–	–
PGE_2_	6AK3	−8.98	2	SER-336 ARG-333	1	TRP-295	MET-137 PRO-55 ASP-99 VAL-110 ALA-335	Dinopristone	−8.24	5	ARG-333 THR-206 TYR-114 SER-336 THR-107	–	–	PHE-140
		−18.63	2	ARG-333 THR-57	–	–	–		−14.82	1	THR-107			
NFĸB	4Q3J	−4.03	3	CYS-149 GLU-233 ARG-232	1	TYR-227	ALA-228 ASP-198 ARG-237	Curcumin	−7.13	4	GLU-184 CYS-149 ARG −237 ASN-240	–	–	PRO-147 ILE-148 LEU-236
		−5.64	2	ARG-26 ALA-151					−8.54	1	ASN-240			
TNF-α	1BKC	−8.06	5	ALA-439 LYS-432 ASN-447 ASN-389 GYL-346	–	–	LEU-348 VAL-402 VAL-440 HIS-444 THR-347	Aspirin	−5.20	4	GLY-349 HIS-405 HIS-409 HIS-415	1	HIS-405	THR-347 LEU-348 GLU-406 ALA-439
		−15.6	1	ARG-357	3	LEU-348 GLY-349 HIS-361	–		−6.13	0	–	–	–	–

Standard inhibitors or activator of pathways are: Omeprazole, phenoxy benzamine, ranitidine, meclofinamate, dinopristone, curcumin and aspirin

Amino acids are: *ARG* Arginine, *ILE* isoleucine, *ASN* asparagine, *TYR* tyrosine, *HIS* histidine, *THR* threonine, *GLU* glutamic acid, *PRO* proline, *PHE* phenylalanine, *VAL* valine, *LYS* lysine, *SER* serine, *CYS* cysteine, *LEU* leucine, *TRP* tryptophan, *ASP* aspartic acid and *ALA* alanine

*AD* AutoDock results, *CD* CDOCKER results

### DPPH free radicals scavenging

DTN at different dose concentrations (200, 66.6, 22.2, 7.4 and 2.46 μg/mL) showed compound anti-oxidant effect 70.13 ± 0.57, 60.27 ± 1.15, 57.83 ± 0.28, 44.32 ± 0.18 and 42 ± 0.46 and ascorbic acid at dose (20 μg/mL) showed 88 ± 0.55 anti-oxidant effect. DTN showed IC_50_ effect at dose concentration on (7.4 μg/mL) and ascorbic acid showed IC_50_ at dose (4.3 μg/mL) respectively (Table 2).

**Table 2:** 2,2-diphenyl-1-picrylhydrazyl (DPPH) free radical scavenging assay of dibutylstannanediyl bis(4-((4-nitrophenyl)amino)-4-oxobut-2-enoate (DTN) and ascorbic acid

Samples	Concentration (μg/mL)	% DPPH inhibition (Mean ± SEM)	IC_**50**_ (μg/mL)
**DTN**	200	70.13 ± 0.57	7.4
	66.6	60.27 ± 1.15	
	22.2	57.83 ± 0.28	
	7.4	44.32 ± 0.18	
	2.46	42 ± 0.46	
**Ascorbic acid**	20	88 ± 0.55	4.3

### *H. pylori* inhibitory effect

DTN anti-*H. pylori* activity against three different strains j63 (cagA-), j196 (cagA-) and j107 (cagA+) were determined by disc diffusion method, metronidazole was used as a positive control. Against strain-I, strain-II and strain-III different concentrations used of DTN and metronidazole were 0.5, 1, 2, 4, 8, 16, and 32 μg/disk and Inhibition diameter (mm) of DTN against strain-I was 2.33 ± 0.66, 3.66 ± 0.33, 4.66 ± 0.33, 6.0 ± 0.57, 10 ± 1.15, 12.66 ± 0.33, 15.33 ± 1 and metronidazole was 3.66 ± 0.33, 4.66 ± 0.33, 5.33 ± 0.66, 7 ± 0.57, 10.33 ± 1.20, 14.66 ± 0.88, 22 ± 1.15. DTN against strain-II was 3 ± 0.57, 3.66 ± 0.66, 5 ± 0.57, 6 ± 1.73, 9.33 ± 0.66, 12.66 ± 0.88, 19 ± 0.57 and metronidazole was 4 ± 0.57, 5 ± 0.57, 5 ± 0.57, 7.33 ± 0.88, 10.33 ± 1.20, 15 ± 1.73, 0.66 ± 2.30. DTN against strain-III was 2.66 ± 0.33, 4 ± 0.57, 4 ± 0.57, 6.33 ± 0.66, 10.33 ± 1.20, 14 ± 0.57, 18.66 ± 0.88 and metronidazole was 4 ± 0.57, 4.66 ± 0.33, 5.66 ± 0.88, 8 ± 1.15, 11.33 ± 0.66, 15.66 ± 0.33, 22.66 ± 0.66 respectively. MIC (μg/ml) of DTN effect against *H. pylori* was 12, 10, 10 and Metronidazole was 4, 6 and 4 (μg/ml) respectively (Table 3).

**Table 3:** Zone of inhibition and minimum inhibitory concentration (MIC) of 2E,2′E) dibutylstannanediyl bis(4-((4-nitrophenyl)amino)-4-oxobut-2-enoate (DTN) against three strains of *H.pylori*, using disk diffusion method

Samples	Zone of Inhibition (mm)							MIC (μg/mL)
	0.5 μg/disk	1 μg/disk	2 μg/disk	4 μg/disk	8 μg/disk	16 μg/disk	32 μg/disk	
**STRAIN I: j63 (cagA-)**								
**DTN**	2.33 ± 0.66	3.66 ± 0.33	4.66 ± 0.33	6.0 ± 0.57	10 ± 1.15	12.66 ± 0.33	15.33 ± 1	12
**Metronidazole**	3.66 ± 0.33	4.66 ± 0.33	5.33 ± 0.66	7 ± 0.57	10.33 ± 1.20	14.66 ± 0.88	22 ± 1.15	4
**STRAIN II: j196 (cagA-)**								
**DTN**	3 ± 0.57	3.66 ± 0.66	5 ± 0.57	6 ± 1.73	9.33 ± 0.66	12.66 ± 0.88	19 ± 0.57	10
**Metronidazole**	4 ± 0.57	5 ± 0.57	5 ± 0.57	7.33 ± 0.88	10.33 ± 1.20	15 ± 1.73	20.66 ± 2.30	6
**STRAIN III: j107 (cagA+)**								
**DTN**	2.66 ± 0.33	4 ± 0.57	4 ± 0.57	6.33 ± 0.66	10.33 ± 1.20	14 ± 0.57	18.66 ± 0.88	10
**Metronidazole**	4 ± 0.57	4.66 ± 0.33	5.66 ± 0.88	8 ± 1.15	11.33 ± 0.66	15.66 ± 0.33	22.66 ± 0.66	4

### Effect on ethanol-induced gastric ulcer

**Fig. 3:**
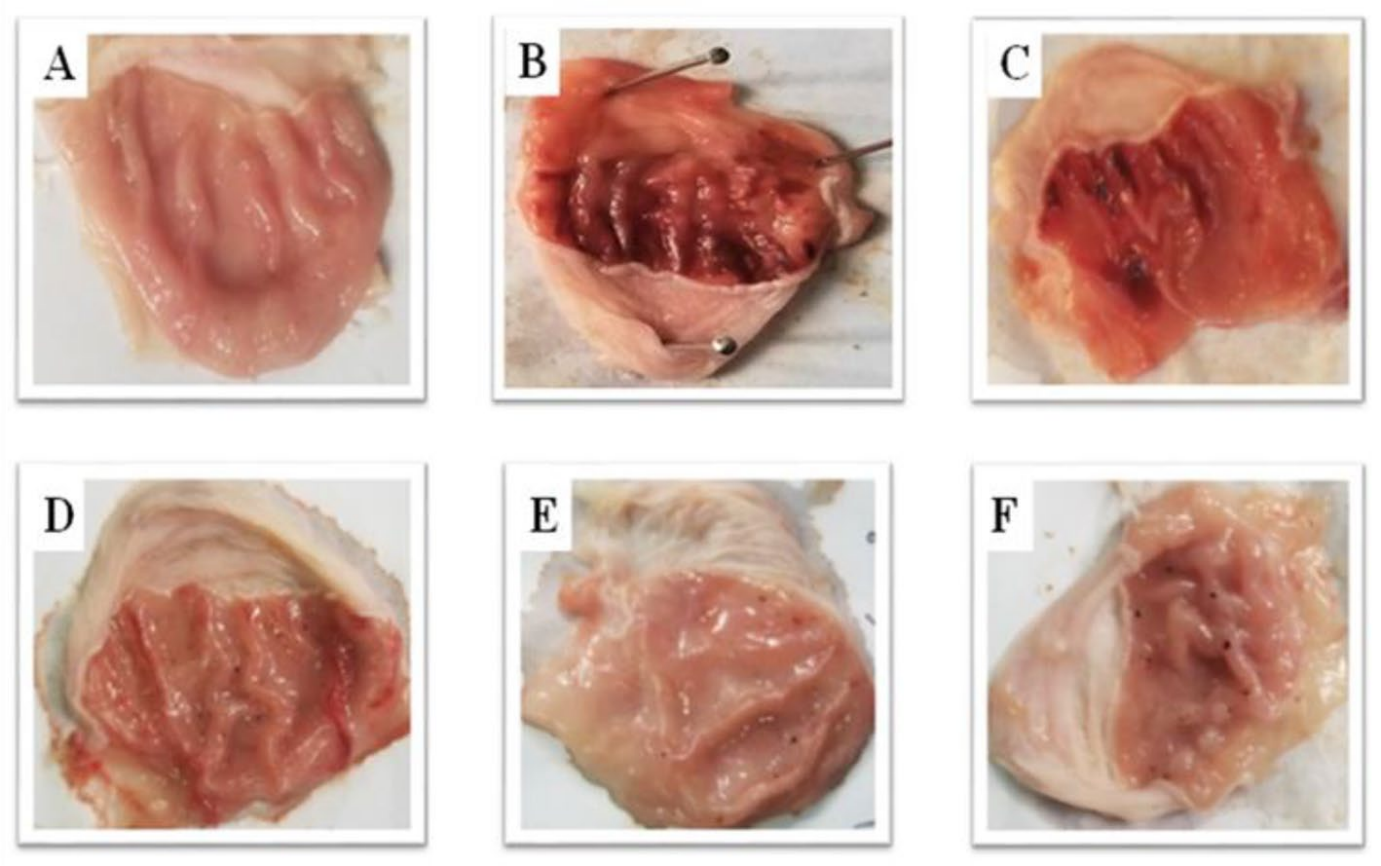
Gross appearance gastric mucosa of rats: **A** pre-treated with saline (10 mL/Kg), **B** treated with absolute ethanol (1 mL/100 g), **C**, **D** and **E** pre-treated with DTN at doses (5, 10 and 30 mg/Kg) and **F** pre-treated with omeprazole (20 mg/Kg)

DTN at (5, 10 and 30 mg/kg) exhibited an antiulcer effect. DTN shows 41, 58 and 90% protective effect at 5, 10 and 30 mg/kg doses with ulcer index scores 10 ± 0.25, 5.9 ± 0.29, 4.2 ± 0.37, 1 ± 0.27 respectively. Omeprazole (20 mg/Kg) exhibited 90% inhibition with ulcer index 1 ± 0.22 as compare to ethanol group respectively (Fig. 3 and Table 4).

**Table 4:** Protective effect of 2E,2′E) dibutylstannanediyl bis(4-(4-nitrophenyl)amino)-4-oxobut-2-enoate (DTN) and omeprazole against ethanol-induced gastric ulcer rats tissues

Treatment (mg/Kg)	Ulcer index ± SEM	% Inhibition
Saline (10 mL/Kg)	0 ± 0	_
Ethanol (1 mL/100 g)	10 ± 0.25^###^	0
DTN (5 mg/Kg) + Ethanol (1 mL/100 g)	5.9 ± 0.29^***^	41
DTN (10 mg/Kg) + Ethanol (1 mL/100 g)	4.2 ± 0.37^***^	58
DTN (30 mg/Kg) + Ethanol (1 mL/100 g)	1 ± 0.27^***^	90
Omeprazole (20 mg/Kg) + Ethanol (1 mL/100 g)	1 ± 0.22^***^	90

Data expressed as mean ± SEM (*n* = 5). One way ANOVA with post-hoc Tukey’s test

^###^*P* < 0.001 vs. saline group, ^***^P < 0.001 vs. ethanol group

### Effect on H^+^/K^+^-ATPase inhibition

**Fig. 4:**
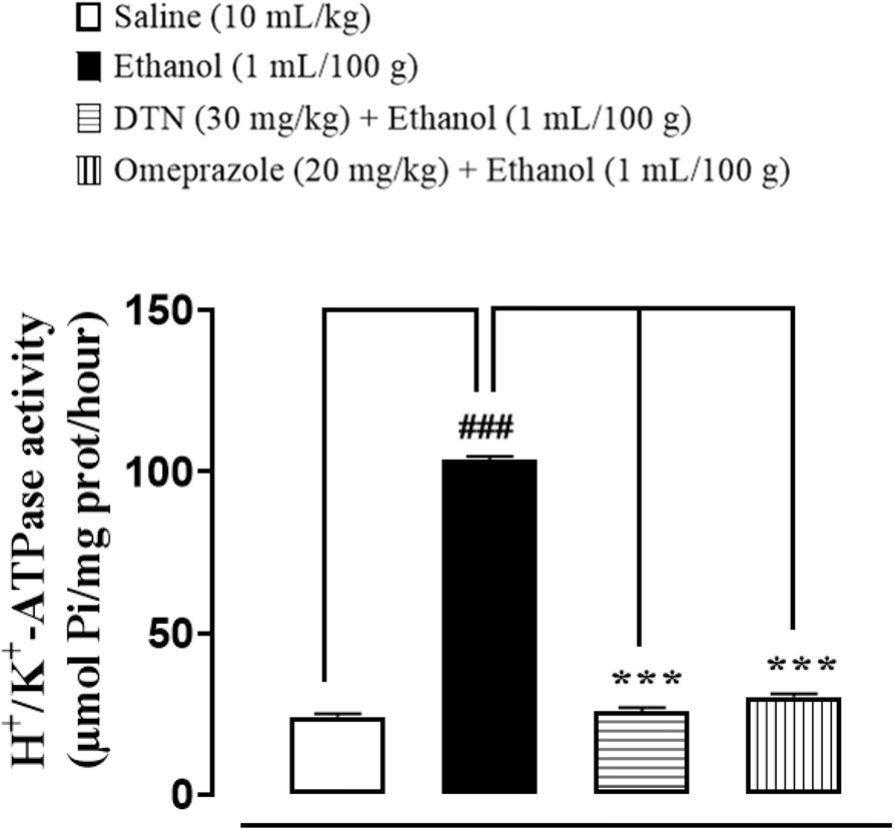
Inhibitory effect of 2E,2′E) dibutylstannanediyl bis(4-((4-nitrophenyl)amino)-4-oxobut-2-enoate (DTN) and omeprazole against H^+^/K^+^-ATPase in ethanol-induced gastric ulcer rats tissues. Values expressed as mean ± SEM (*n* = 5). One way ANOVA with post-hoc Tukey’s test. ^###^*P* < 0.001 vs. saline group, ^***^P < 0.001 vs. ethanol group

In saline group (10 mL/Kg) H^+^/K^+^-ATPase levels was 24.19 ± 0.43 (μmol Pi/mg prot/hour). In ethanol group (1 mL/100 g) the level was significantly increased to 103.8 ± 0.44 (μmol Pi/mg prot/hour) as compared to saline group. In treatment group DTN (30 mg/Kg) expression of H^+^/K^+^-ATPase is 25.96 ± 0.44 and omeprazole (20 mg/Kg) was 30.29 ± 0.44 significantly decreased as compared to ethanol group (Fig. 4).

### Effect on oxidative stress markers

**Fig. 5:**
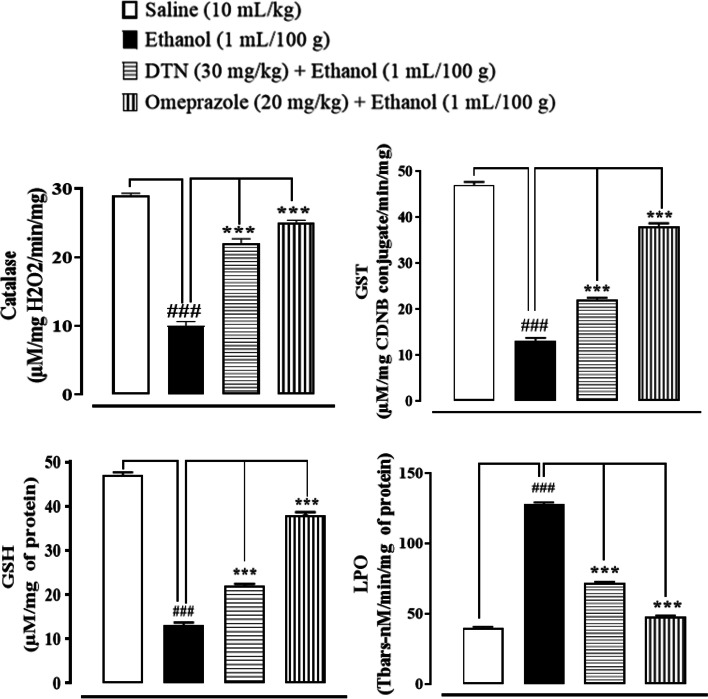
Effect of 2E,2′E) dibutylstannanediyl bis(4-(4-nitrophenyl)amino)-4-oxobut-2-enoate (DTN) and omeprazole against catalase, glutathione sulfotransferases (GST), glutathione (GSH), and lipid peroxide (LPO) in ethanol-induced gastric ulcer rats tissues. Data expressed as mean ± SEM (*n* = 5). One-way ANOVA, with post-hoc Tukey^,^s test. ^###^*P* < 0.001 vs. saline group, ^***^P < 0.001 vs. ethanol group

In saline group (10 mL/Kg), Catalase, GST, GSH and LPO levels were 70 ± 0.31 μmoles H_2_0_2_ /min/mg, 66.2 ± 0.66 CDNB conjugate/min/mg, 47 ± 0.63 μmoles/min/mg and 40 ± 0.70 (Tbars-nM/min/mg) respectively. In ethanol group (1 mL/100 g), Catalase, GST, GSH and LPO levels were 10 ± 0.63 μmoles H_2_0_2_/min/mg, 17 ± 0.63 CDNB conjugate/min/mg, 13 ± 0.70 μmoles/min/mg and 128 ± 1.2 (Tbars-nM/min/mg). In DTN treated group (30 mg/Kg) stomach tissues catalase, GST, GSH and LPO levels were 22 ± 0.70 μmoles H_2_0_2_ /min/mg, 48 ± 0.70 CDNB conjugate/min/mg, 22 ± 0.44 μmoles/min/mg and 72 ± 0.70 Tbars-nM/min/mg respectively. In Omeprazole (20 mg/Kg) treated group stomach tissues Catalase, GST, GSH and LPO levels were 25 ± 0.63 μmoles H_2_0_2_ /min/mg, 61 ± 0.63 CDNB conjugate/min/mg, 38 ± 0.63 μmoles/min/mg and 48 ± 0.70 Tbars-nM/min/mg respectively (Fig. 5).

### Histopathological examination

**Fig. 6:**
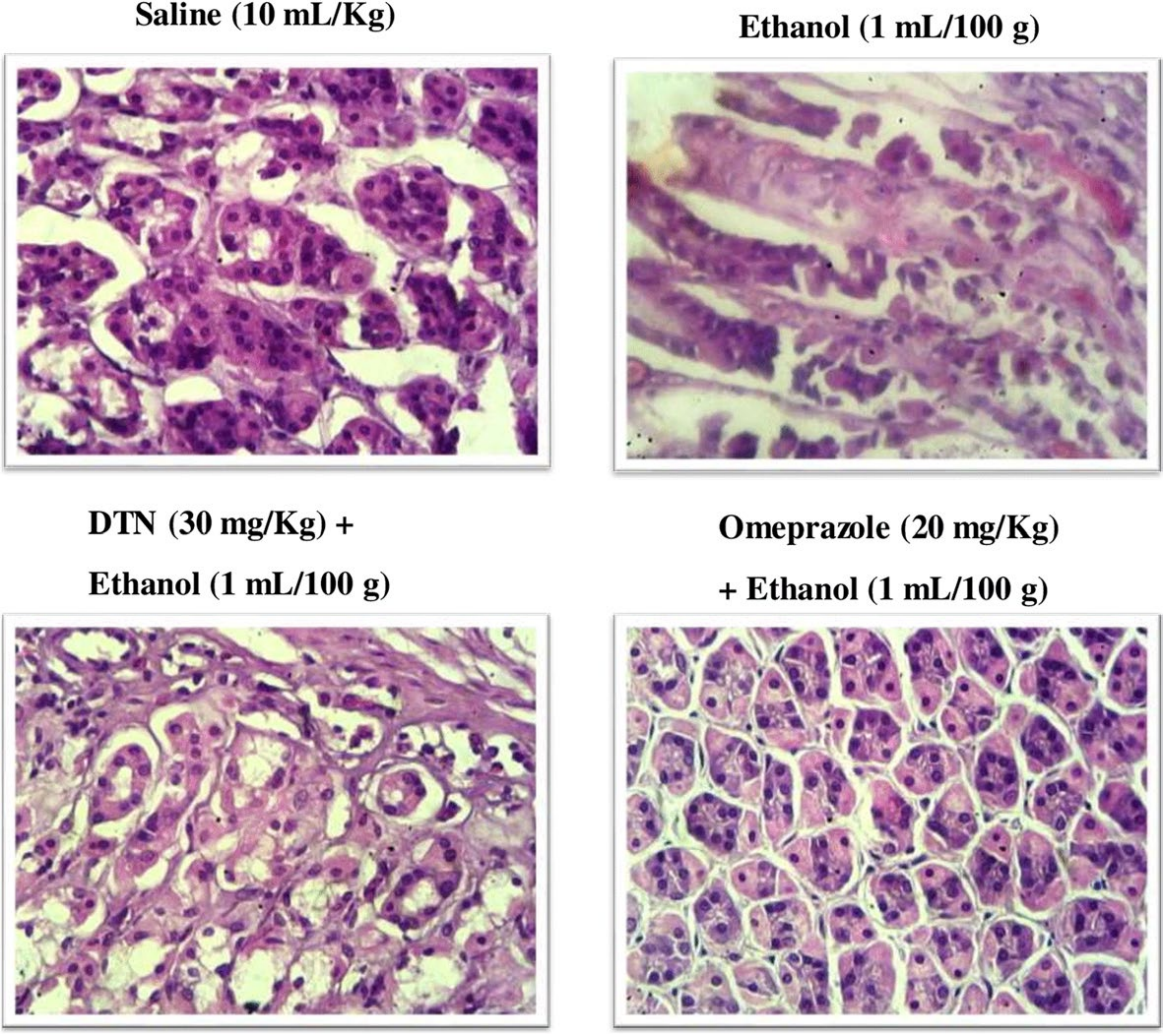
Histopathological examaination, hematoxylin and eosin (H&E) stained slides represent effect of 2E,2′E) dibutylstannanediyl bis(4-(4-nitrophenyl)amino)-4-oxobut-2-enoate (DTN) and omeprazole in ethanol-induced gastric ulcer rats tissues

Saline (10 mL/Kg) group revealed normal stomach tissues architecture without any pathological changes. Ethanol (1 mL/100 g) treated tissues exhibited severe gastric damaged with vacuolation and disruption of morphological cell boundaries. DTN (30 mg/Kg) and omeprazole (20 mg/Kg) treated gastric tissues revealed the regeneration and restoration of stomach cells with mild degeneration respectively (Fig. 6).

### IHC analysis

**Fig. 7:**
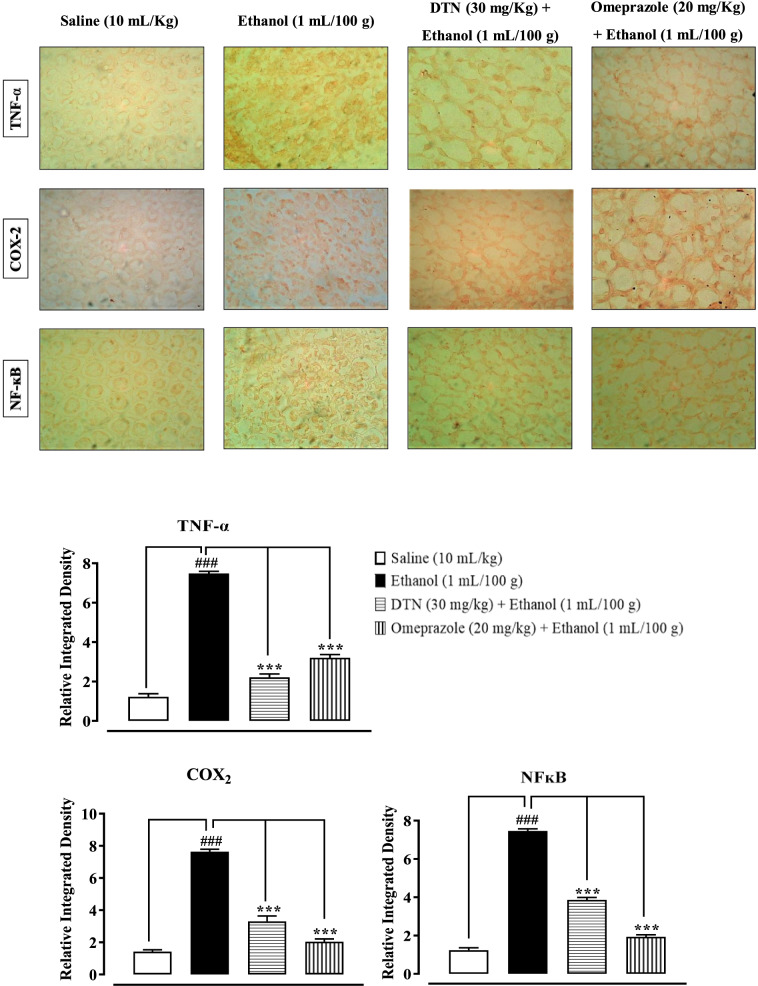
Slides and bar charts represents effect of 2E,2′E) dibutylstannanediyl bis(4-(4-nitrophenyl)amino)-4-oxobut-2-enoate (DTN) and omeprazole against tumor necrosis factor alpha (TNF-α), cyclooxygenase-2 (COX_2_) and tumor necrosis factor kappa-B (p-NFĸB) expression in ethanol-induced gastric ulcer rats tissues, using immunohistochemistry technique

IHC of gastric tissues revealed that ethanol (1 mL/100 g) treated group markedly upregulated inflammatory markers COX-2, p-NFκB and TNF-α expression. Vacuolation, necrotic cells and disruption of morphological cell boundaries were found in disease group. DTN (30 mg/Kg) and omeprazole (20 mg/Kg) group’s downregulated COX-2, p-NFκB and TNF-α expression (Fig. 7).

### Effect on inflammatory markers

**Fig. 8:**
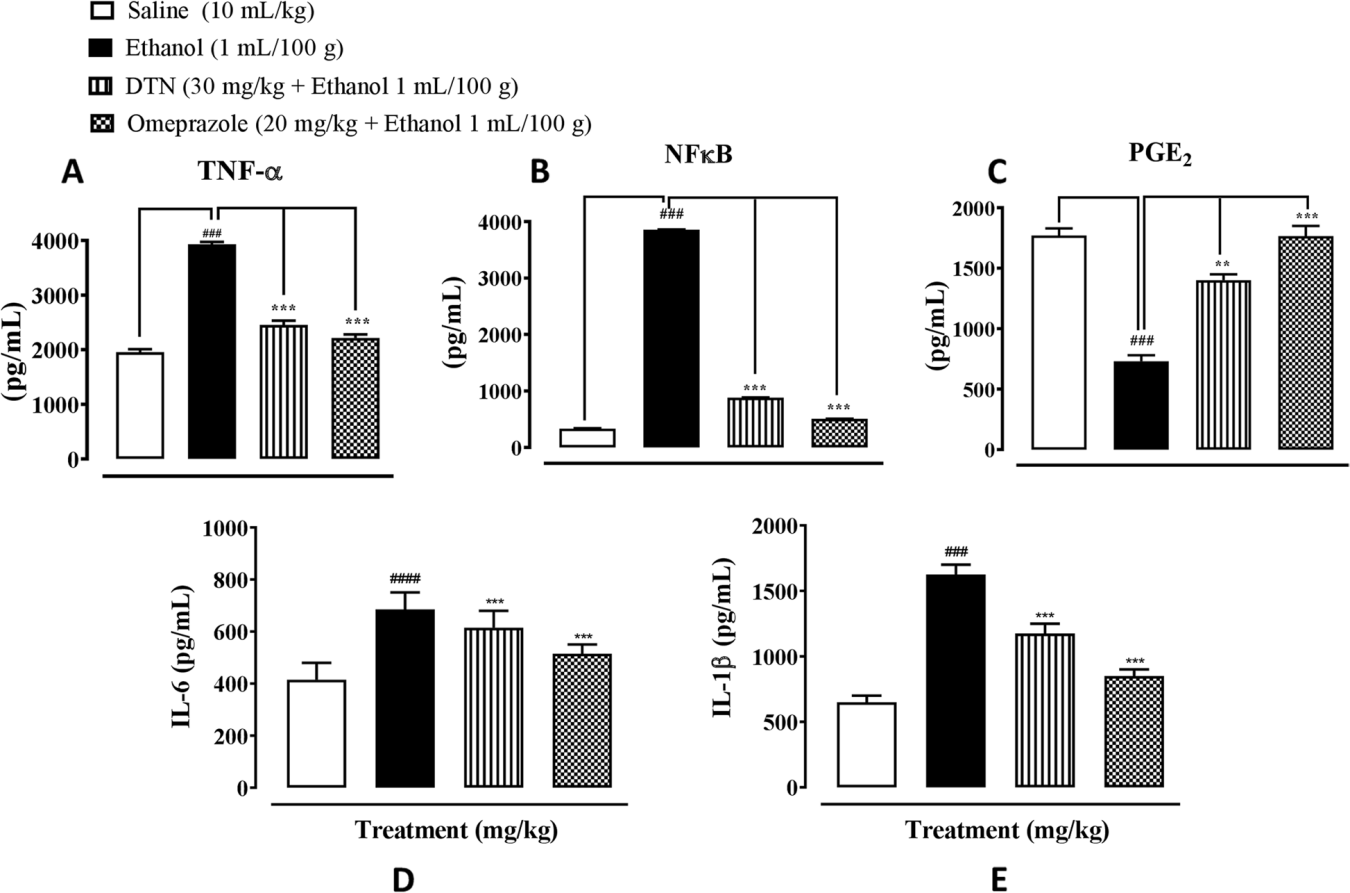
**A**, **B**, **C**, **D** and **E** Represent the effect of 2E,2′E) dibutylstannanediyl bis(4-(4-nitrophenyl)amino)-4-oxobut-2-enoate (DTN) and omeprazole against tumor necrosis factor alpha (TNF-α), phosphorylated nuclear factor kappa B (p-NFĸB), prostaglandins E2 (PGE_2_), interleukin 6 (IL-6) and interleukin 1 beta (IL-1β) in ethanol-induced gastric ulcer rats tissues, using enzyme linked immunosorbent assay technique (ELISA). Data expressed as mean ± SEM (*n* = 5). One-way ANOVA, with post-hoc Tukey’s test. ^###^*P* < 0.001 vs. saline group, ^**^P < 0.01, ^***^P < 0.001 vs. ethanol group

In gastric tissues, saline group (10 mL/Kg), p-NF-ĸB, TNF-α, PGE2, IL-6 and IL-1 β levels were 330 ± 9.52, 1955 ± 55 and 1770 ± 60 pg/mg, 415 ± 1.1 and 650 ± 0.89 pg/mL respectively. In ethanol group (1 mL/100 g) p-NFĸB, TNF-α, IL-6 and IL-1 β levels were 3855 ± 3.70, 3931 ± 40,685 ± 0.77 and 1625 ± 0.85 significantly increased and PGE_2_ level was 730 ± 50 pg/mg significantly decreased as compare to saline group. In DTN treated group (30 mg/Kg) p-NFĸB, TNF-α, IL-6 and IL-1 β levels were 880 ± 3.64, 2455 ± 75, 615 ± 1.1 and 1175 ± 0.34 pg/mg significantly decreased and PGE_2_ level was 1400 ± 50 pg/mg significantly increased as compare ethanol group. In omeprazole treated group (20 mg/Kg) p-NFĸB, TNF-α, IL-6 and IL-1 β levels were 505 ± 3.5, 2215 ± 65, 515 ± 0.99 and 850 ± 0.66 pg/mg significantly decreased and PGE_2_, 1765 ± 85 pg/mg significantly increased as compare to ethanol group respectively (Fig. 8).

### Western blot findings

**Fig. 9:**
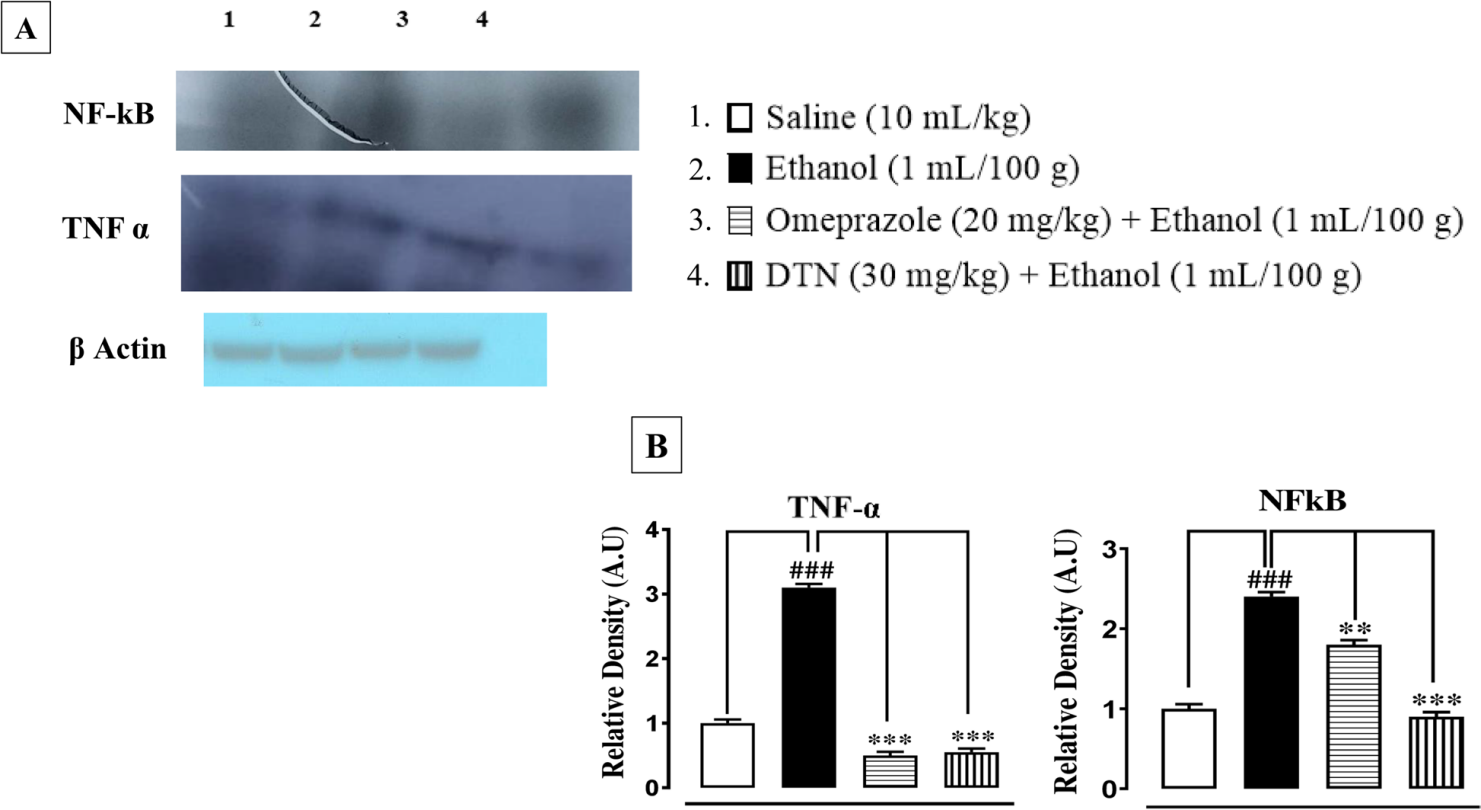
**A** Bands and **B** bar charts represent Inhibitory effect of 2E,2′E) dibutylstannanediyl bis(4-(4-nitrophenyl)amino)-4-oxobut-2-enoate (DTN) and omeprazole against tumor necrosis factor alpha (TNF-α) and necrosis factor kappa B (p-NFĸB) in gastric ulcer tissues of ethanol treated rats, using western blot analysis. Data expressed as mean ± SEM (*n* = 5). One-way ANOVA, with post-hoc Tukey’s test. ^###^*P* < 0.001 vs. saline group, ^**^P < 0.01, ^***^P < 0.001 vs. ethanol group

In ethanol group (1 mL/100 g), TNF-α and p-NF-ĸB expression in the gastric mucosa region were increased. In treatment group DTN (30 mg/Kg) and omeprazole (20 mg/Kg) suppressed the elevated expression of TNF-α and p-NF-ĸB (Fig. 9).

### Quantification of m-RNA level

**Fig. 10:**
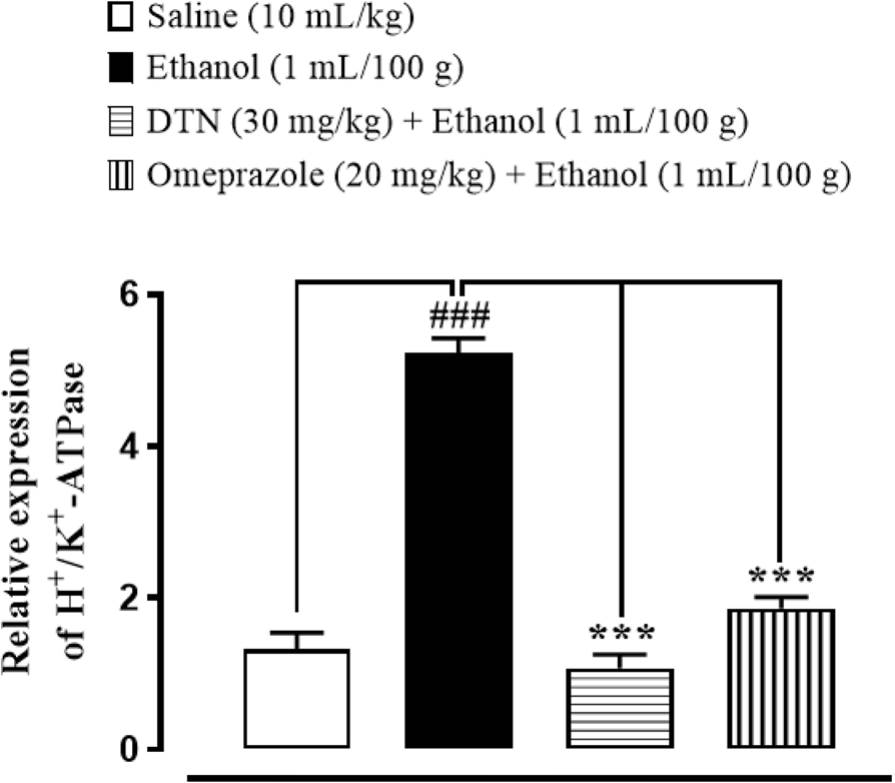
Inhibitory effect of 2E,2′E) dibutylstannanediyl bis(4-(4-nitrophenyl)amino)-4-oxobut-2-enoate (DTN) and omeprazole against messenger ribonucleic acid (mRNA) expression in gastric ethanol-induced gastric ulcer rats tissues, using reverse transcriptase polymerase chain reaction (RT-PCR). Values expressed as mean ± SEM (*n* = 5). One-way ANOVA with post-hoc Tukey’s test. ^###^*P* < 0.001 vs. saline group, ^***^P < 0.001 vs. ethanol group

RT-PCR determined fold expression of H^+^/K^+^-ATPase in ethanol-induced gastric ulcer. In ethanol treated group expression of H^+^/K^+^-ATPase mRNA levels increased. DTN (30 mg/Kg) and omeprazole (20 mg/Kg) decreased H^+^/K^+^-ATPase mRNA levels (Fig. 10).

### Acute toxicity

The DTN did not caused any mortality up to 400 mg/Kg.

## Discussion

The current study confirmed that newly synthesized test compound DTN exhibits anti-ulcer effect (mediated via anti-*H. pylori,* H^+^/K^+^-ATPase inhibition, anti-oxidant and anti-inflammatory pathways), exploring its therapeutic potential in gastric ulcer management. Characterization of a newly synthesized compound was performed to confirm the final product using different confirmatory methods.^13^CNMR techniques were used for further confirmation of the final synthesized product organotin (IV) complex. Identification tests results proved that the final product was confirmed and the bands of functional group of the desired product lies in range [16]. A compound anti-oxidant assay was performed by DPPH method. A newly prepared compound has strong free radicals scavenging effect and becomes similar to positive control ascorbic acid [15].

Molecular docking is the key method of structured virtual screening and it is still a very active area in research [11]. The interaction strength of π-π is described as the stability of the comparable structural complex to hydrogen bonding strength [26]. In the ground state condition, π-π interaction does not affect the functionality of the active site, but will result in a 20-30 fold reduction in the rate permanent of chemical activity hydrophobic interactions also increase ligand affiliation against target proteins [27]. The ligands and protein targets complexes were assessed by the atomic contact energy (ACE) value, H. bond, π-π interaction and hydrophobic interaction in AutoDock and by ACE value, H.bond in CDOCKER respectively. The DTN compound showed significant binding to various protein targets in our present study. Order of atomic contact energy values (kcal/mol) against targeted proteins through AutoDock and C-DOCKER with high to low (ACE-value) are: NFĸB > COX_1_ > H_2_ > TNF-α > PGE_2_ > H^+^/K^+^-ATPase pump > M_1_ > COX_2_ and NFĸB > COX_1_ > H^+^/K^+^-ATPase pump > H_2_ > COX_2_ > TNF-α > M_1_ > PGE_2_ respectively. To confirm the docking analysis performed with AutoDock, the docking results were crosschecked using CDOCKER. All docking conformations were visualized using DSV so as to ensure the ligands were docked into the defined binding pocket. The ACE against H^+^/K^+^-ATPase pump, COX_1_ and NFkB proteins were almost the same in both AutoDock and CDOCKER software but there were some differences between ACE against M_1_,H_2_, COX_2_, PGE_2_ and TNF-α targets having lower binding energies than AutoDock. The lower value of CDocker energy gives the best binding affinity of the ligand to the receptor protein [28].

In the in-vitro conformational analysis, *H. pylori* is the main risk factor for gastric ulcer disease [3]. Organotin (IV) complex previously reported good anti-bacterial inhibitory activity [16]. DTN possesses anti-bacterial activity as it inhibits *H. pylori* bacteria, mainly responsible for gastric ulceration [16]. Anti *H. pylori* activity was examined through a zone of inhibition and minimum inhibitory concentration. DTN showed an anti *H. pylori* effect through the zone of inhibition and MIC against three different clinical strains.

Many studies demonstrate that alcohol has an essential role in gastric injury and ulceration [5]. Ethanol-induced ulcer model is used to investigate and diagnose gastric ulcer pathogenesis and study the gastrointestinal effect of various medicines and natural products against ulcer [5, 29]. Ethanol-induced gastric ulcer is due to the increased production of inflammatory cells and reactive oxygen species that accumulate and further trigger oxidative damage [6, 7]. We have observed that oxidative stress was particularly severe in the ethanol group, which is consistent with previous studies [30]. From a macroscopic gross view of gastric mucosa and microscopic (H&E) slides observations, DTN pretreatment effectively eliminates gastric ulcers. All treatment groups indicated a substantial dose-dependent reduction in the area of gastric ulcers. These results are ongoing agreement with earlier studies that evaluated the gastro-protection of various synthetics recognition of the gastrointestinal role of DTN in compounds and ethanol-induced gastric ulcers [31].

Ethanol increases gastric mucosa expression of H+/K + -ATPase and increases stomach acid and pepsin secretions, which are the most important causes in gastric mucosa injury. Prior to ethanol treatment, stomach hemorrhagic lesions reduce mucosal blood flow, resulting in K+ pumps in and Na + pumps out, causing gastric acid to leap [4]. In the clinic, inhibiting the activity of the H+/K + -ATPase becomes effective treatment for stomach ulcers [32]. The model group’s H+/K + -ATPase activity was significantly increased by ethanol stimulation in this investigation. The abnormal activation of H+/K + -ATPase is suppressed by DTN treatment in a dose-dependent manner. Our molecular docking results for H+/K + -ATPase also verified this. We discovered that DTN might be used as a potential therapy for gastric ulcers by inhibiting H+/K + -ATPase activity, which resulted in a consistent reduction in gastric acid volume and acidity. The pathogenic process of stomach ulcer has been connected to oxidative stress [33]. Oxidative stress has been linked to ulcers on the stomach mucosa. Free radicals that are extremely cytotoxic are produced in various ways [6]. Because lipids are significant elements of cell membranes, oxygen-derived free radicals were found to combine with them to generate lipid peroxides, which caused widespread damage [34]. According to previous research, increased free radical production accounts for the membrane damage seen in pathological experiments, as demonstrated by increased lipid peroxidation LPO, notably TBARS. The body’s anti-oxidant defense system can scavenge free radicals produced from oxygen, including anti-oxidants such as GSH, GST and catalase. Depleting cellular GSH and GST and decreased catalase activity may make recovery from ethanol-induced stomach oxidative injury more difficult [35]. Ethanol exposure produces significant decreases in GSH, GST and catalase activities and an increase in LPO TBARS levels, according to the results of this study. On the other hand, DTN pretreatment resulted in significant increases in GSH, GST, and catalase levels, as well as a decrease in LPO TBARS, indicating its antioxidant capability and demonstrating that the molecule possesses gastro-protective effects against ethanol-model ulcers. There is a well-known connection between inflammation and ethanol-induced stomach ulcer damage [36].

IHC staining of inflammation causing cytokines of ethanol-induced gastric ulcer was further experimentally examined. It revealed over expression of p-NF-ĸB, TNF-α and COX_2_ in ethanol treated stomach compared to control. While pretreatment of DTN at (30 mg/Kg) dose decreases the expression of p-NF-ĸB, TNF-α and COX_2_ compared to the ulcer group [37, 38]. PGE2 plays a crucial role as a mediator. It was well-known for its ability to protect the gastric mucosa and repair gastric ulcers [38]. Stomach ulceration is caused by decreased PGE2 levels in the gastric mucosa, which aggravates pre-existing gastric ulcers [37]. In a previous investigation, ethanol treatment was shown to lower PGE2 levels [39]. In ethanol gastric ulcers model rats, the effects of our current work are consistent with some previous studies, which have shown that DTN compound can defeat hemorrhagic gastric mucosal lesions by regulating PGE2 production, preventing the accumulation of inflammatory cells and increasing anti-oxidant enzyme activity [37]. As a result, the gastro-protective effect of DTN may be linked to the protection of stomach prostaglandin.

Ethanol induces the inflammatory markers that initiates macrophages produce vast volumes of pro-inflammatory cytokines like TNF-α, p-NF-ĸB, IL-6 and IL-1β which promote the accumulation of neutrophils in the inflammation site, leading to the breakdown and the destruction of the mucosal barrier and ROS activates p-NF-ĸB through IĸB phosphorylation [34, 39]. These pro-inflammatory cytokines produce an increased quantity of oxygen-derived free radicals, thereby facilitating the formation of stomach ulcers. Our findings outcomes were in agreements with those earlier work [35]. While ethanol exposure ulcer raised p-NFĸB, TNF-α, IL-6 and IL-1β levels associated with control rats. In contrastively, treatment with DTN reversed the elevated levels of p-NFĸB, TNF-α, IL-6 and IL-1β. Even though these cytokines up to reach at normal levels (30 mg/Kg) under DTN pretreatment, the reduction of the p-NFĸB level in DTN-pretreated rats might be a result of ROS scavenging ability of DTN, which shows its anti-inflammatory effect on ethanol-exposure gastric ulcer in rats.

Western blot findings provide evidence that DTN has an anti-inflammatory effect through reduced expression of TNF-α and p-NF-ĸB. Both proteins are involved in the recruitment of inflammatory mediators, in the previous study it was revealed that the inflammatory mediator of TNF-α and p-NF-ĸB downregulated in ethanol-induced ulcerated tissue of the stomach [39]. The RT-PCR technique provides close evidence and further confirmation of the targeted H^+^/K^+^-ATPase pump inhibition pathway that m-RNA levels of H^+^/K^+^-ATPase was determined in ethanol group. While treatment with DTN and omeprazole significantly reduced the level of H^+^/K^+^-ATPase compared to the ethanol group [40]. It was confirmed from different experimental studies that DTN (30 mg/Kg) has gastro protective effect mediated through, occupied targets receptors with favorable (ACE-values), anti-*H.pylori,* H^+^/K^+^-ATPase inhibition, anti-oxidant and anti-inflammatory pathways. Due to multi effective property, it may reduce polypharmacy, be economically cost-effective, decrease medication error, drug-drug interactions and be effective in ulcer disease management.

## Conclusion

This study reveals that the newly synthesized compound DTN, possesses binding energy values − 4.03 to − 10.64 Kcal/mol against selected targets. DTN exhibits an anti-ulcer effect, mediated via anti-*H.pylori,* H^+^/K^+^-ATPase inhibition, anti-oxidant and anti-inflammatory pathways demonstrate its therapeutic potential in management of gastric ulcer.

## Supplementary Information

**Additional file 1: Figure S1.** (A) and (B) represents 2D-interactions of 2E,2′E) dibutylstannanediyl bis(4-(4-nitrophenyl)amino)-4-oxobut-2-enoate (DTN) and omeprazole with hydrogen potassium atipase pump (H^+^/K^+^-ATPase) evaluated through Biovia Discovery Studio Visualizer (DSV) 2016. **Figure S2.** (A) and (B) represents 2D-interactions of 2E,2′E) dibutylstannanediyl bis(4-(4-nitrophenyl)amino)-4-oxobut-2-enoate (DTN) and phenoxy benzamine with muscarinic receptor (M_1_) respectively, evaluated through Biovia Discovery Studio Visualizer (DSV) 2016. **Figure S3.** (A) and (B) represents 2D-interactions of 2E,2′E) dibutylstannanediyl bis(4-(4- nitrophenyl)amino)-4-oxobut-2-enoate (DTN) and ranitidine with histamine receptor (H_2_) respectively, evaluated through Biovia Discovery Studio Visualizer (DSV) 2016. **Figure S4.** (A) and (B) represents 2D-interactions of 2E,2′E) dibutylstannanediyl bis(4-(4- nitrophenyl)amino)-4-oxobut-2-enoate (DTN) and aspirin with cyclooxiginase-1 (COX_1_) respectively, evaluated through Biovia Discovery Studio Visualizer (DSV) 2016. **Figure S5.** (A) and (B) represents 2D-interactions of 2E,2′E) dibutylstannanediyl bis(4-(4-nitrophenyl)amino)-4-oxobut-2-enoate (DTN) and meclofenamate with cyclooxiginase-2 (COX_2_) respectively, evaluated through Biovia Discovery Studio Visualizer (DSV) 2016. **Figure S6.** (A) and (B) represents 2D-interactions of 2E,2′E) dibutylstannanediyl bis(4-(4-nitrophenyl)amino)-4-oxobut-2-enoate (DTN) and dinopristone with prostaglandin-E_2_ (PGE_2_) respectively, evaluated through Biovia Discovery Studio Visualizer (DSV) 2016. **Figure S7.** (A) and (B) represents 2D-interactions of 2E,2′E) dibutylstannanediyl bis(4-(4- nitrophenyl)amino)-4-oxobut-2-enoate (DTN) and curcumin with nuclear factor kappa B (NFĸB) respectively, evaluated through Biovia Discovery Studio Visualizer (DSV) 2016. **Figure S8.** (A) and (B) represents 2D-interactions of 2E,2′E) dibutylstannanediyl bis(4-(4-nitrophenyl)amino)-4-oxobut-2-enoate (DTN) and aspirin with tumor necrosis factor alpha (TNF- α) respectively, evaluated through Biovia Discovery Studio Visualizer (DSV) 2016. **Figure S1A.** (A) and (B) represents 2D-interactions of 2E,2′E) dibutylstannanediyl bis(4-(4-nitrophenyl)amino)-4-oxobut-2-enoate (DTN) and Omeprazole with hydrogen potassium ATPase (H^+^/K^+^-ATPase) evaluated through Biovia Discovery Studio Visualizer (DSV) 2016. **Figure S2A.** (A) and (B) represents 2D-interactions of 2E,2′E) dibutylstannanediyl bis(4-(4-nitrophenyl)amino)-4-oxobut-2-enoate (DTN) and Phenoxy-benzamine with Muscarinic receptor (M_1_) evaluated through Biovia Discovery Studio Visualizer (DSV) 2016. **Figure S3A.** (A) and (B) represents 2D-interactions of 2E,2′E) dibutylstannanediyl bis(4-(4-nitrophenyl)amino)-4-oxobut-2-enoate (DTN) and Ranitidine with histaminergic receptor (H_2_), evaluated through Biovia Discovery Studio Visualizer (DSV) 2016. **Figure S4A.** (A) and (B) represents 2D-interactions of 2E,2′E) dibutylstannanediyl bis(4-(4-nitrophenyl)amino)-4-oxobut-2-enoate (DTN) and Aspirin with cyclooxygenase-1 (COX_1_), evaluated through Biovia Discovery Studio Visualizer (DSV) 2016. **Figure S5A.** (A) and (B) represents 2D-interactions of 2E,2′E) dibutylstannanediyl bis(4-(4-nitrophenyl)amino)-4-oxobut-2-enoate (DTN) and Meclofinamate with cyclooxygenase-2 (COX_2_) evaluated through Biovia Discovery Studio Visualizer (DSV) 2016. **Figure S6A.** (A) and (B) represents 2D-interactions of 2E,2′E) dibutylstannanediyl bis(4-(4-nitrophenyl) amino)-4-oxobut-2-enoate (DTN) and Dinopristone with prostaglandin-E2 (PGE_2_), evaluated through Biovia Discovery Studio Visualizer (DSV) 2016. **Figure S7A.** (A) and (B) represents 2D-interactions of 2E,2′E) dibutylstannanediyl bis(4-(4-nitrophenyl)amino)-4-oxobut-2-enoate (DTN) and Curcumin with Nuclear Factor kappa-B (NFκB) evaluated through Biovia Discovery Studio Visualizer (DSV) 2016. **Figure S8A.** (A) and (B) represents 2D-interactions of 2E,2′E) dibutylstannanediyl bis(4-(4-nitrophenyl) amino)-4-oxobut-2-enoate (DTN) and aspirin with Tumor necrosis factor alpha (TNF-α) evaluated through Biovia Discovery Studio Visualizer (DSV) 2016.

**Additional file 2.**

**Additional file 3.**

**Additional file 4.**

**Additional file 5.**

## Data Availability

All data is provided in the manuscript.
