# Supplementary material for: Pharmacological evaluation of newly synthesized organotin IV complex for antiulcer potential
Source: BMC Pharmacol Toxicol. 2022 Jul 29;23:58. doi: 10.1186/s40360-022-00596-0 (PMC9335977; doi:10.1186/s40360-022-00596-0)
Supplement: Supplementary file 1 — Additional file 1: Figure S1. (A) and (B) represents 2D-interactions of 2E,2′E) dibutylstannanediyl bis(4-(4-nitrophenyl)amino)-4-oxobut-2-enoate (DTN) and omeprazole with hydrogen potassium atipase pump (H+/K+-ATPase) evaluated through Biovia Discovery Studio Visualizer (DSV) 2016. Figure S2. (A) and (B) represents 2D-interactions of 2E,2′E) dibutylstannanediyl bis(4-(4-nitrophenyl)amino)-4-oxobut-2-enoate (DTN) and phenoxy benzamine with muscarinic receptor (M1) respectively, evaluated through Biovia Discovery Studio Visualizer (DSV) 2016. Figure S3. (A) and (B) represents 2D-interactions of 2E,2′E) dibutylstannanediyl bis(4-(4- nitrophenyl)amino)-4-oxobut-2-enoate (DTN) and ranitidine with histamine receptor (H2) respectively, evaluated through Biovia Discovery Studio Visualizer (DSV) 2016. Figure S4. (A) and (B) represents 2D-interactions of 2E,2′E) dibutylstannanediyl bis(4-(4- nitrophenyl)amino)-4-oxobut-2-enoate (DTN) and aspirin with cyclooxiginase-1 (COX1) respectively, evaluated through Biovia Discovery Studio Visualizer (DSV) 2016. Figure S5. (A) and (B) represents 2D-interactions of 2E,2′E) dibutylstannanediyl bis(4-(4-nitrophenyl)amino)-4-oxobut-2-enoate (DTN) and meclofenamate with cyclooxiginase-2 (COX2) respectively, evaluated through Biovia Discovery Studio Visualizer (DSV) 2016. Figure S6. (A) and (B) represents 2D-interactions of 2E,2′E) dibutylstannanediyl bis(4-(4-nitrophenyl)amino)-4-oxobut-2-enoate (DTN) and dinopristone with prostaglandin-E2 (PGE2) respectively, evaluated through Biovia Discovery Studio Visualizer (DSV) 2016. Figure S7. (A) and (B) represents 2D-interactions of 2E,2′E) dibutylstannanediyl bis(4-(4- nitrophenyl)amino)-4-oxobut-2-enoate (DTN) and curcumin with nuclear factor kappa B (NFĸB) respectively, evaluated through Biovia Discovery Studio Visualizer (DSV) 2016. Figure S8. (A) and (B) represents 2D-interactions of 2E,2′E) dibutylstannanediyl bis(4-(4-nitrophenyl)amino)-4-oxobut-2-enoate (DTN) and aspirin with tumor necrosi [file 40360_2022_596_MOESM1_ESM.docx]

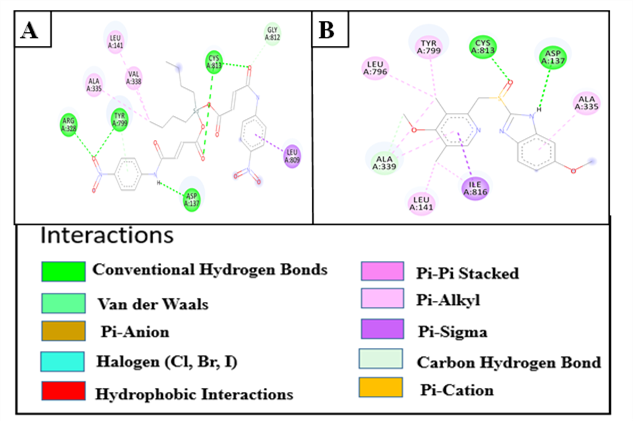


**Figure S1. (A)** and (**B)** represents 2D-interactions of 2E,2'E) dibutylstannanediyl bis(4-(4- nitrophenyl)amino)-4-oxobut-2-enoate (DTN) and omeprazole with hydrogen potassium atipase pump (H^+^/K^+^-ATPase) evaluated through Biovia Discovery Studio Visualizer (DSV) 2016.
