# Supplementary figures and images for: Pharmacological evaluation of newly synthesized organotin IV complex for antiulcer potential
Source: BMC Pharmacol Toxicol. 2022 Jul 29;23:58. doi: 10.1186/s40360-022-00596-0 (PMC9335977; doi:10.1186/s40360-022-00596-0)

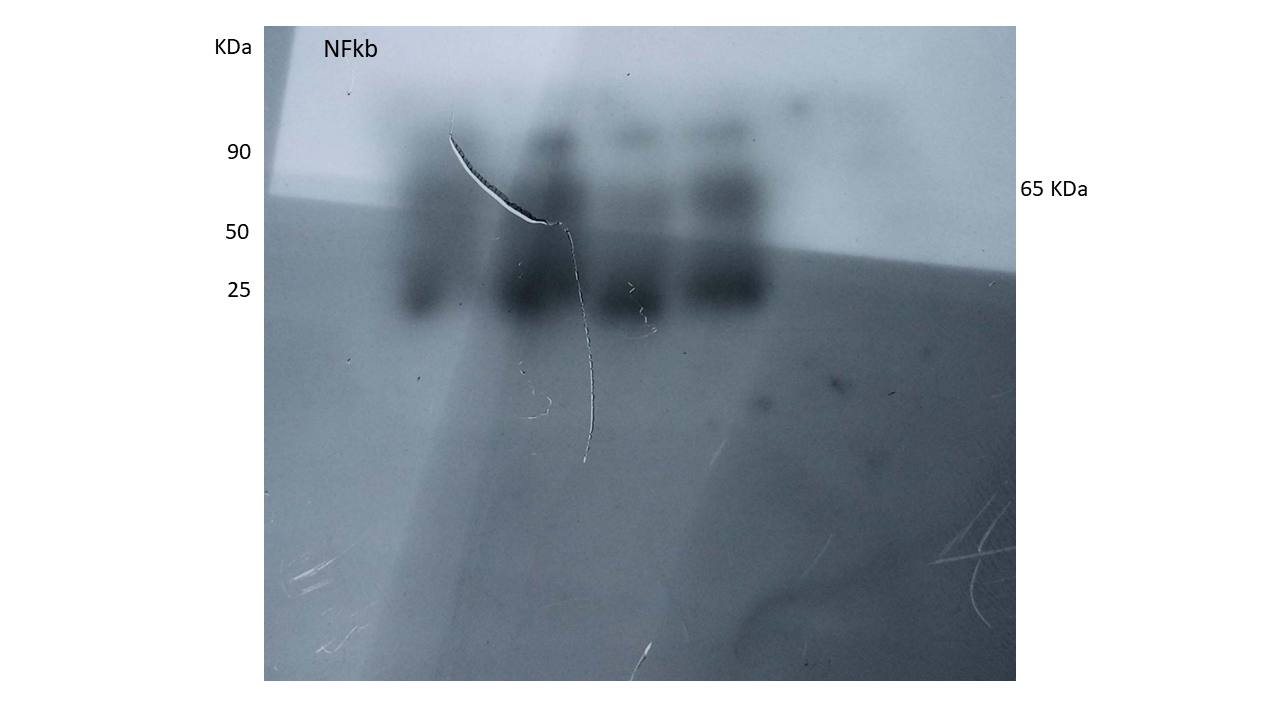

Supplement: Supplementary file 2 — Additional file 2. [file 40360_2022_596_MOESM2_ESM.tif]

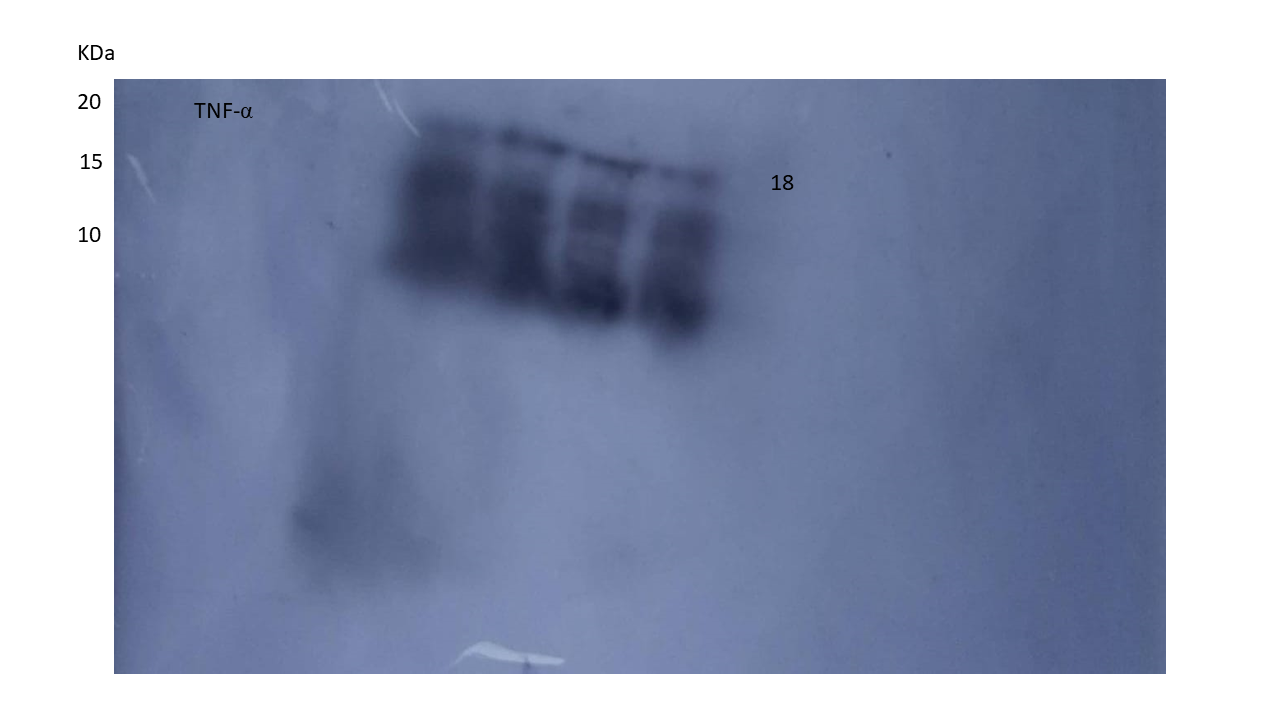

Supplement: Supplementary file 3 — Additional file 3. [file 40360_2022_596_MOESM3_ESM.tif]

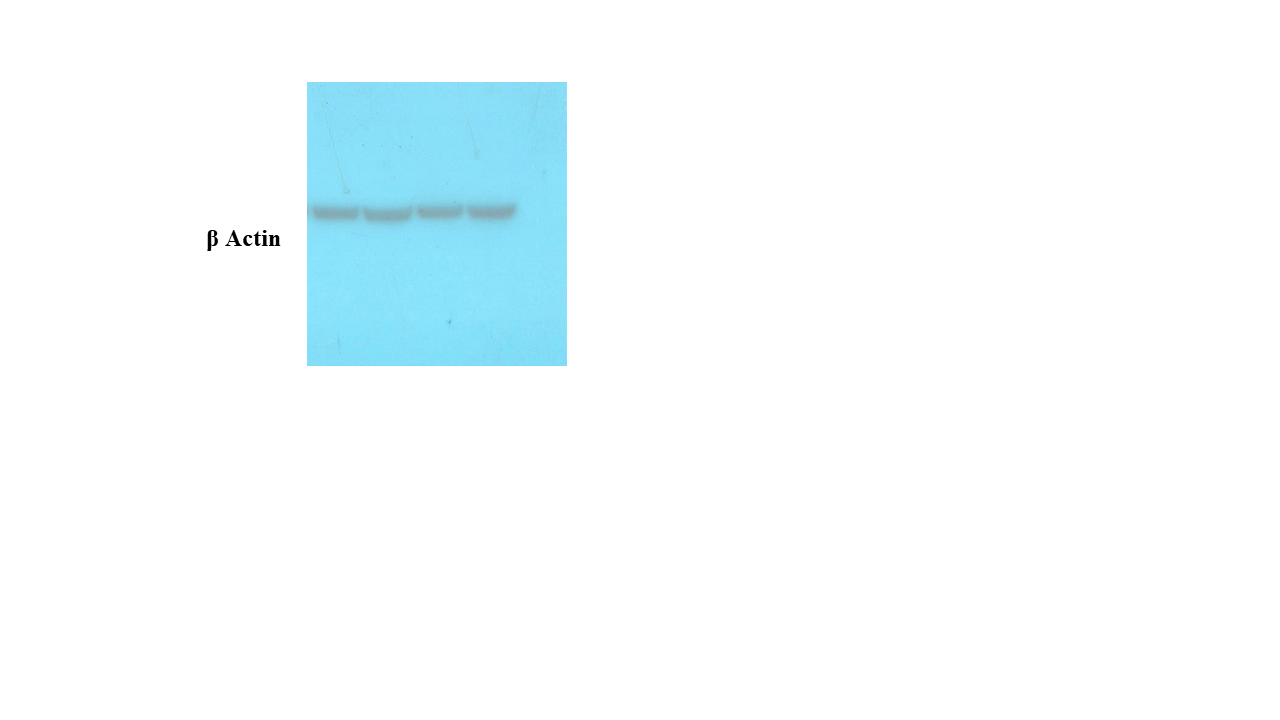

Supplement: Supplementary file 4 — Additional file 4. [file 40360_2022_596_MOESM4_ESM.tif]

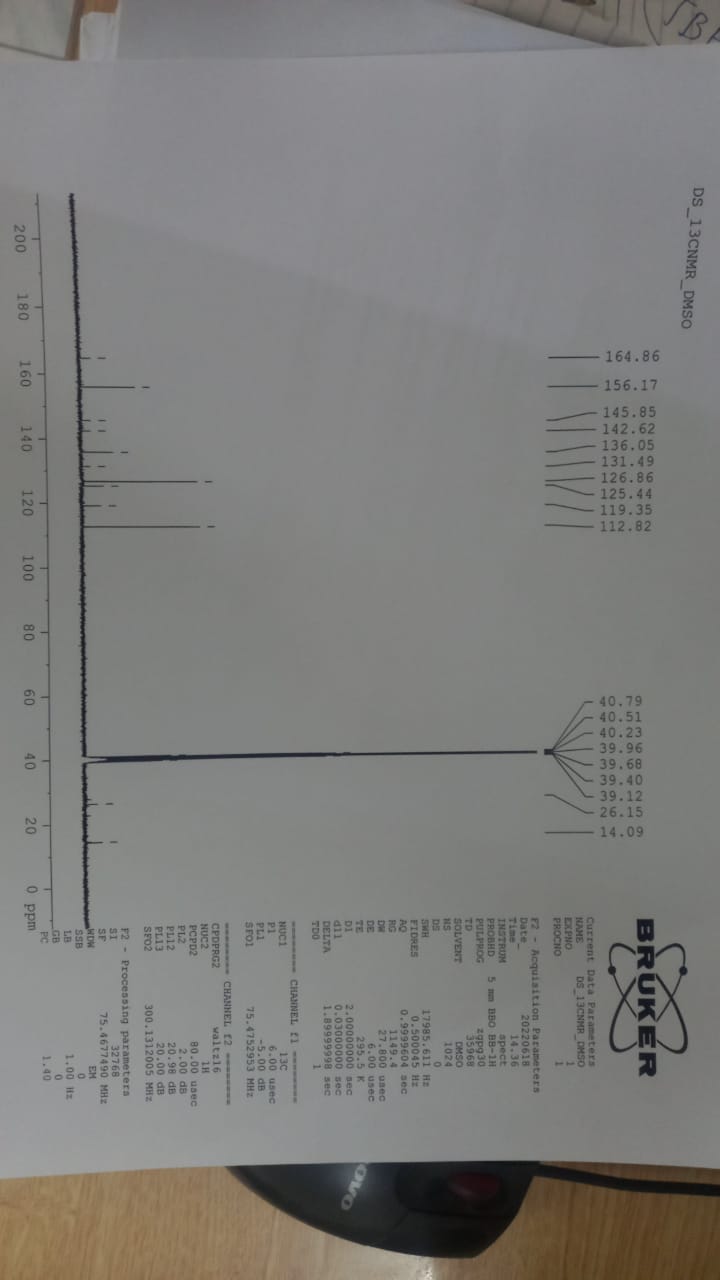

Supplement: Supplementary file 5 — Additional file 5. [file 40360_2022_596_MOESM5_ESM.jpeg]
